# Integrated Physics-Informed Machine Learning Framework for Structural Damage Detection, Localization, and Severity Classification

**DOI:** 10.3390/s26154976

**Published:** 2026-08-05

**Authors:** Zixin Wang, Mohammad R. Jahanshahi

**Affiliations:** 1 Department of Civil and Environmental Engineering, University of Illinois Urbana-Champaign, Urbana, IL 61801, USA; 2 Lyles School of Civil and Construction Engineering, Purdue University, 550 Stadium Mall Drive, West Lafayette, IN 47907, USA; jahansha@purdue.edu; 3 Elmore Family School of Electrical and Computer Engineering, Purdue University, 610 Purdue Mall, West Lafayette, IN 47907, USA

**Keywords:** structural health monitoring, structural damage identification, physics-informed machine learning, hybrid digital twins, domain adaptation, deep learning

## Abstract

Structural health monitoring (SHM) plays a critical role in the early identification and assessment of structural damage, thereby enhancing the safety and reliability of civil infrastructure. Structural damage identification generally encompasses three key tasks: damage detection, localization, and quantification. While extensive research has been conducted on each of these tasks individually, relatively few studies have integrated all three components into a unified framework for comprehensive structural condition assessment. Physics-based approaches require an accurate finite element model (FEM), which is often difficult to calibrate to accurately represent the behavior of the actual structure. In contrast, data-driven approaches rely on sufficient labeled data collected from the actual structure, which is likewise challenging to acquire in practice. To address these limitations, this work proposes an integrated hierarchical physics-informed domain adaptation (I-HierPhyDA) framework that performs damage detection, localization, and severity classification in a hierarchical manner. The proposed framework bridges the gap between the reduced-order FEM and the higher-fidelity FEM by generating vibration signatures that are consistent across both domains. Furthermore, the proposed framework enables structural damage localization without requiring damage-state data from the target domain during training, while damage severity classification is performed using transductive domain adaptation with unlabeled damaged-state data from the target domain. The proposed framework is systematically evaluated using the numerical ASCE benchmark models under structural uncertainties and measurement noise. The results demonstrate that the proposed approach achieves accurate structural damage detection and localization. For structural damage severity classification, it achieves the highest mean accuracy and Macro-F1 score while exhibiting the lowest standard deviations for both metrics among the baseline and ablation methods, demonstrating its effectiveness for comprehensive structural condition assessment. Future work will focus on experimentally validating the proposed approach using measured data from laboratory or field structures.

## 1. Introduction

Structural systems are widely used across various engineering disciplines, including civil [1,2], mechanical [3], and aerospace engineering [4]. Over time, harsh environmental conditions and operational loads can lead to the degradation or failure of structural systems, posing significant threats to their safety, reliability, and serviceability. For example, civil infrastructure may experience structural damage due to natural hazards such as earthquakes, hurricanes, and typhoons [5]. Furthermore, a substantial portion of existing civil infrastructure is more than 50 years old, making age-related deterioration an increasingly critical concern [6]. Therefore, it is essential to develop effective structural health monitoring (SHM) techniques capable of identifying structural damage at an early stage, thereby enabling timely maintenance, retrofit, and informed decision-making to ensure the continued safety and reliability of structural systems.

Structural damage identification methods can be broadly classified into physics-based and data-driven approaches. Physics-based methods can be further categorized into modal parameter-based approaches and model updating-based approaches. Since global structural damage will alter the modal parameters, the damage detection is performed by tracking the change of modal parameters, including natural frequencies [7,8,9,10,11,12,13,14,15,16], mode shapes [17,18,19,20], modal curvatures [21,22,23], modal strain energy [22,24], frequency response functions [25,26,27], transmissibility functions [28], and nonlinear characteristics [29,30]. Moreover, quite a few researchers have combined several modal parameters together to collect sufficient modal information in damage detection, such as the combination of natural frequencies and mode shapes [31,32,33,34,35,36,37,38,39,40], the combination of natural frequencies, mode shapes, and damping ratios [41,42,43]. Although numerous damage detection applications using modal parameter-based methods have been reported, they have been demonstrated to be insufficient to estimate a robust set of damage-sensitive features. Structural uncertainties and measurement noise may influence the modal parameters, and the true damage information may be concealed by the uncertainties. Recent studies have demonstrated the successful application of modal-analysis-based SHM to aerospace structures through noise-robust modal parameter identification and full-scale multi-input multi-output (MIMO) ground vibration testing, highlighting the transferability of vibration-based SHM techniques across engineering disciplines. However, these studies also demonstrate that aerospace structures often require more sophisticated experimental setups, including MIMO excitation and dense sensor instrumentation, to accurately identify closely spaced vibration modes, reflecting the increased experimental complexity associated with full-scale aerospace applications [44,45].

Model updating-based approaches rely on a numerical representation of the structure, such as a finite element model (FEM), and infer damage by updating model parameters, such as mass and stiffness, to achieve agreement between simulated and measured structural responses [46]. However, due to noisy measurements, structural uncertainties, and limited sensing data, model updating is often formulated as an ill-conditioned inverse problem, making it challenging to obtain an accurate FEM that faithfully represents the actual structure [47]. Furthermore, structural uncertainties arising from environmental and operational variations, as well as uncertainties in material properties and boundary conditions, are typically not considered, which may adversely affect the accuracy of damage identification [48]. Consequently, the existing physics-based approaches for structural damage identification using model updating are limited to simple and small-scale structural systems due to their high computational costs of analyzing complex, large-scale structures [49].

Representative full-scale SHM studies have demonstrated the importance of integrating experimental modal identification, multisensor measurements, and FEM calibration for reliable structural health assessment. For example, Serlenga et al. [50] conducted full-scale bridge monitoring using multisensor measurements and experimental modal identification to establish a reference state for FEM calibration, while Kim et al. [51] conducted ambient and vehicle-induced vibration tests on a full-scale steel truss bridge with artificial damage, providing a benchmark for vibration-based damage identification. These studies highlight the importance of accurate modal parameter identification and field measurements while emphasizing the need to account for measurement noise, environmental variability, sensor limitations, and operational conditions when bridging numerical models and actual structures.

Recently, artificial intelligence and deep learning have demonstrated remarkable capabilities in extracting complex patterns from large-scale data and achieving strong generalization across a wide range of SHM tasks, including structural damage identification [52], structural system identification [53], structural response estimation [54], and structural load prediction [55]. Data-driven approaches based on deep learning can generally be categorized into two groups: supervised learning methods and unsupervised learning methods. Unsupervised learning methods learn patterns exclusively from unlabeled data [56]. Various unsupervised learning techniques have been employed for anomaly detection in structural systems, such as autoencoders (AEs) [57,58], one-class support vector machines [59], principal component analysis [60], deep Boltzmann machines [61], and generative adversarial networks [62]. Among these techniques, AEs have been extensively employed for anomaly detection in safety-critical systems. By training AEs exclusively on healthy-state data, anomalies are identified when the reconstruction error exceeds a predefined threshold. Although unsupervised learning methods have demonstrated strong performance in anomaly detection, their ability to localize and quantify defects within a system remains limited.

On the other hand, supervised learning methods require sufficient labeled data to establish a mapping between measured structural responses and corresponding damage states. Various supervised learning techniques have been utilized for structural damage identification, including convolutional neural networks (CNNs) [63], support vector machines [64], and multilayer perceptrons [65]. Although these methods are capable of localizing and quantifying structural damage once properly trained, the availability of labeled data representing diverse damage scenarios in actual structures remains extremely limited. This limitation arises because it is generally impractical and costly to intentionally damage real structures for the purpose of data collection. To alleviate this challenge, FEMs can be employed to generate large amounts of synthetic data for training supervised learning models. However, calibrating an FEM to accurately represent an actual structure is often an ill-posed inverse problem. As a result, discrepancies between the FEM and the real structure, together with measurement noise and structural uncertainties, introduce a domain gap between the synthetic training data from the FEM (i.e., source domain) and the measured data from the actual structure (i.e., target domain). Consequently, models trained on FEM-generated data may experience significant performance degradation when deployed on real structures, since supervised learning generally assumes that the training and testing data follow the same underlying data distribution [66].

Structural damage identification generally comprises three key tasks: damage detection [67,68], damage localization [69,70], and damage quantification [71,72]. While extensive research has been conducted on each of these tasks individually, relatively few studies have integrated all three within a unified framework to enable comprehensive structural health assessment. Physics-informed machine learning has gained significant attention in recent years because it improves model generalizability [73] and enables effective training with limited data by incorporating physical constraints into the learning process [74]. Physics-informed neural networks are typically formulated by integrating data-driven and physics-based loss functions. For instance, the governing equations of motion can be incorporated into the physics-based loss to enforce physical consistency during training. However, such implementations face challenges in real-world applications, as it is generally difficult to obtain the full structural state across all degrees of freedom (DOFs), including displacement, velocity, and acceleration responses. Furthermore, the analytical model employed in the governing equations may not accurately represent the actual structure due to uncertainties in structural parameters, particularly structural damping, which is often difficult to identify and calibrate in practice.

In this work, we propose an integrated physics-informed machine learning framework for structural damage detection, localization, and severity classification. The proposed framework addresses these three tasks in a hierarchical manner, enabling a comprehensive assessment of the structural health condition. Unlike conventional physics-informed approaches that require access to the full structural state, the proposed framework relies solely on acceleration responses and does not require displacement or velocity measurements. Furthermore, it enables the model to learn vibration characteristics that are shared between the reduced-order FEM (source domain) and the higher-fidelity FEM (target domain), thereby reducing the domain discrepancy between the two domains. As a result, the framework enables structural damage localization without requiring damage-state data from the target domain during training, while damage severity classification is performed using transductive domain adaptation with unlabeled damaged-state data from the target domain. The proposed framework was systematically evaluated using the numerical ASCE benchmark models while accounting for structural uncertainties and measurement noise. The results demonstrate that the proposed framework achieves 100% accuracy in structural damage detection and localization. For structural damage severity classification, it outperforms the baseline and ablation methods by achieving the highest mean accuracy and mean Macro-F1 score, while exhibiting the lowest standard deviations for both metrics, demonstrating its superior accuracy and robustness.

## 2. Methodology

### 2.1. Overview

**Figure 1:**
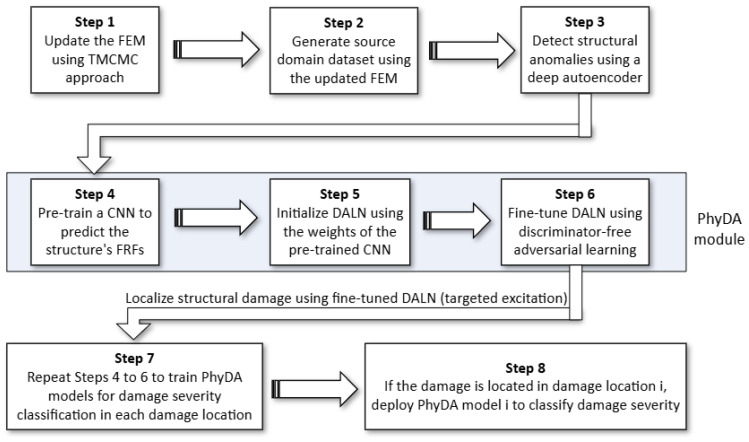
An overview of the integrated hierarchical physics-informed domain adaptation (I-HierPhyDA) framework for structural damage detection, localization, and severity classification.

In the proposed integrated hierarchical physics-informed domain adaptation (I-HierPhyDA) framework, the structure is excited by impact forces applied at various locations to enable active sensing. Because impulsive excitation possesses a broad frequency spectrum, multiple structural vibration modes can be excited simultaneously, providing rich modal information for structural damage identification. Existing studies have employed automatic driving vehicles [75] and unmanned aerial vehicles (UAVs) [76] to generate impact forces for bridge inspection. Therefore, active impact excitation on actual structures can be feasibly implemented using mobile robots or robotic arms [77]. The continuous wavelet transforms (CWTs) of the acceleration responses are employed for structural damage identification, as they capture both temporal and spectral characteristics of the signals and provide superior time–frequency resolution for analyzing nonstationary structural responses [78]. The FEM used to generate source-domain data is updated using the transitional Markov chain Monte Carlo (TMCMC) method [79]. The model updating process using the TMCMC method is presented in Appendix A. Structural damage detection is first performed using unsupervised deep autoencoders to determine whether the structure is damaged. If damage is detected, the framework proceeds to the next stage, where a physics-informed domain adaptation (PhyDA) model trained for damage localization is used to identify the damaged location. Subsequently, PhyDA models trained separately for damage severity classification at each potential damage location are activated according to the localization results to estimate the corresponding damage severity. An overview of the proposed framework is shown in Figure 1.

### 2.2. Structural Damage Detection

**Figure 2:**
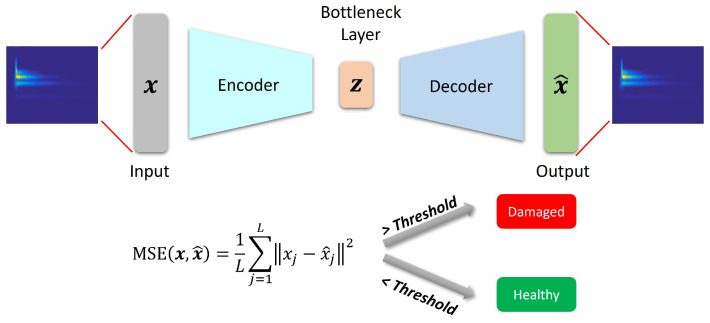
Unsupervised damage detection using CAEs.

As the preliminary step of the proposed framework, structural damage detection (i.e., structural anomaly detection) is performed using convolutional autoencoders (CAEs) and information fusion to determine whether the structure is healthy or damaged [57]. As an unsupervised learning technique, AEs are widely used for dimensionality reduction, feature extraction, and noise suppression. AEs typically comprise two components: an encoder and a decoder, as shown in Figure 2. The encoder projects the input data into a lower-dimensional latent space, whereas the decoder reconstructs the original input from the latent representations. Through learning compact latent representations from unlabeled data, AEs can effectively capture salient features and underlying characteristics of the input signals.

CAEs, a variant of AEs, employ convolutional kernels with shared weights to automatically extract features from input data. Consider a CAE with a single convolutional layer in the encoder and a single transposed convolutional layer in the decoder. The latent representation of the *i*th feature map, $z^{\left(i\right)}$, and the corresponding reconstructed input, $\hat{x}$, can then be formulated as Equations (1) and (2), respectively:

(1) $$z^{\left(i\right)}=f\left(x\right)=\sigma_{f}\left(x*W_{f}^{\left(i\right)}+b_{f}^{\left(i\right)}\right),$$

(2) $$\hat{x}=g\left(z\right)=\sigma_{g}\left(\sum_{z\in Z}z^{\left(i\right)}*{\tilde{W}}_{g}^{\left(i\right)}+b_{g}\right),$$

where x and $\hat{x}$ denote the input and reconstructed output data, respectively, while z represents the latent representation. $W_{f}$ and $W_{g}$ are the encoder and decoder weight matrices, respectively, and $b_{f}$ and $b_{g}$ are the corresponding bias vectors. $\sigma_{f}$ and $\sigma_{g}$ denote the encoder and decoder activation functions, respectively. The operator ∗ represents 2D convolution, *Z* denotes the set of latent feature maps, and ${\tilde{W}}_{g}^{\left(i\right)}$ denotes the decoder kernel obtained by flipping $W_{g}^{\left(i\right)}$ along both spatial dimensions, i.e., ${\tilde{W}}_{g}^{\left(i\right)}\left(p,q\right)=W_{g}^{\left(i\right)}\left(P-p+1,\;Q-q+1\right)$, where P×Q denotes the kernel size. The detailed architecture of the CAE used for structural damage detection is summarized in Table 1.

Because the output of a CAE is designed to reconstruct the input data, the training process seeks to minimize the discrepancy between the input x and the reconstructed output $\hat{x}$. Accordingly, the mean squared error (MSE) is used as the loss function for network training:

(3) $$L\left(W_{f},W_{g},b_{f},b_{g}\right)=\mathrm{MSE}\left(x,\hat{x}\right)=\frac{1}{L}\sum_{j=1}^{L}{\left(x_{j}-{\hat{x}}_{j}\right)}^{2},$$

where *j* denotes the *j*th input feature, and *L* is the total number of input features.

The CAE is trained solely on healthy-state data collected from the higher-fidelity FEM, as such data are relatively easier to obtain than damage-state data in practice. Consequently, healthy-state responses can be reconstructed with low reconstruction errors. Since the CAE does not learn the characteristics of damaged structural responses during training, damage-state data are expected to yield larger reconstruction errors. Accordingly, when the reconstruction error, measured by the MSE, exceeds a predefined threshold *τ*, the structure can be identified as potentially damaged, which can be formulated as

(4) $$\Theta =\left\{\begin{array}{cc} 1, & \mathrm{if}\;\mathrm{MSE}(x,\hat{x})>\tau ,\\ 0, & \mathrm{otherwise}, \end{array}\right.$$

where Θ denotes the structural damage state, with 1 corresponding to the damaged condition and 0 corresponding to the healthy condition.

To improve the robustness of structural damage detection in the presence of structural uncertainties and measurement noise, an information fusion strategy, referred to as sensor fusion, is proposed. Consider a structure instrumented with *M* accelerometers and excited by hammer impacts at *N* different locations. The trained CAE generates a prediction of the structural condition based on the response measured by each accelerometer for each excitation location. Consequently, a total of *M* × *N* individual predictions are obtained. Sensor fusion integrates the predictions from multiple sensors for a given excitation location. Specifically, if more than *m* out of *M* sensors classify the structure as damaged, the overall prediction is determined to be damaged; otherwise, the structure is classified as healthy. This decision rule can be expressed as

(5) $$\Psi_{j}=\left\{\begin{array}{cc} 1, & \mathrm{if}\;\sum_{i=1}^{M}\Theta_{ij}>m,\\ 0, & \mathrm{otherwise}, \end{array}\right.$$

where $\Theta_{ij}$ denotes the prediction obtained from the *i*th sensor under the *j*th impact location. $\Psi_{j}$ represents the fused prediction for the *j*th impact location, obtained by aggregating the predictions from the *M* sensors using a threshold *m*. When the number of sensors predicting damage is equal to M/2 (e.g., an 8–8 split for a 16-sensor configuration), no majority is achieved. Therefore, the majority-voting criterion in Equation (5) is not satisfied, and the structure is classified as healthy.

### 2.3. Structural Damage Localization

If the structure is identified as damaged, then the next phase is to localize the damage using the physics-informed domain adaptation (PhyDA) approach [80]. A large amount of synthetic data representing various damage scenarios can be generated using the updated reduced-order FEM. However, the updated reduced-order FEM may still differ from the higher-fidelity FEM due to model simplifications and uncertainties in material properties and boundary conditions. As a result, a domain gap may exist between the updated reduced-order FEM (i.e., the source domain) and the higher-fidelity FEM (i.e., the target domain), leading to degraded performance of supervised learning models trained on the source-domain data when applied to the target domain. Domain adaptation, a branch of transfer learning, aims to transfer the knowledge learned from a source domain to improve performance on a target domain. In particular, feature-based unsupervised domain adaptation seeks to learn domain-invariant feature representations that are shared across both domains by leveraging labeled data from the source domain and unlabeled data from the target domain.

Adversarial domain adaptation typically consists of three components: a feature extractor G(·), a domain discriminator D(·), and a damage detector C(·). The feature extractor is responsible for extracting informative features from the input data, while the domain discriminator is trained to distinguish whether the extracted features originate from the source domain or the target domain. The feature extractor and the domain discriminator are optimized in an adversarial manner using labeled source-domain data and unlabeled target-domain data to learn domain-invariant feature representations shared by both domains. Meanwhile, the damage detector is trained using the labeled source-domain data to learn domain knowledge relevant to structural damage identification. In this work, the discriminator-free adversarial learning network (DALN) [81] is employed to mitigate the domain gap between the reduced-order FEM and the higher-fidelity FEM. Unlike conventional adversarial domain adaptation methods, DALN does not require a separate domain discriminator. Instead, the damage detector serves as the domain discriminator. As a task-specific classifier, the damage detector inherently possesses the ability to distinguish between the source and target domains and can therefore be directly utilized for domain discrimination. Accordingly, the domain discriminator can be reformulated as $D\left(\cdot \right)={∥C\left(\cdot \right)∥}_{*}$, where ${∥\cdot ∥}_{*}$ denotes the nuclear norm, thereby establishing the discriminator-free adversarial learning paradigm of DALN. The detailed architectures of the feature extractor and the damage detector in DALN are summarized in Table 2. The training objective of DALN is formulated as a min–max game between the Nuclear-norm 1-Wasserstein discrepancy loss $L_{nwd}$ and the supervised classification loss $L_{cls}$:

(6) $$L_{nwd}\left(x_{s},x_{t}\right)=\frac{1}{N_{s}}\sum_{i=1}^{N_{s}}{∥C\left(G(x_{s}^{\left(i\right)})\right)∥}_{*}-\frac{1}{N_{t}}\sum_{j=1}^{N_{t}}{∥C\left(G(x_{t}^{\left(j\right)})\right)∥}_{*},$$

(7) $$L_{cls}\left(x_{s},y_{s}\right)=\frac{1}{N_{s}}\sum_{i=1}^{N_{s}}L_{ce}\left(C\left(G(x_{s}^{\left(i\right)})\right),y_{s}^{\left(i\right)}\right)=-\frac{1}{N_{s}}\sum_{i=1}^{N_{s}}\sum_{k=1}^{K}1_{\left[k=y_{s}^{\left(i\right)}\right]}log{\left[C\left(G(x_{s}^{\left(i\right)})\right)\right]}_{k},$$

(8) $$\min_{C,G}\left\{L_{cls}\left(x_{s},y_{s}\right)+\lambda \max_{C}L_{nwd}\left(x_{s},x_{t}\right)\right\},$$

where $N_{s}$ and $N_{t}$ denote the numbers of labeled source-domain samples $x_{s}^{\left(i\right)}$ with corresponding labels $y_{s}^{\left(i\right)}$ and unlabeled target-domain samples $x_{t}^{\left(j\right)}$, respectively, and *K* is the number of damage categories. $1_{\left[k=y_{s}^{\left(i\right)}\right]}$ is an indicator function that takes a value of 1 when $k=y_{s}^{\left(i\right)}$ and 0 otherwise.

The DALN is optimized using two objectives. First, the feature extractor *G* and classifier *C* jointly minimize the supervised source-domain classification loss $L_{cls}$. Second, *G* and *C* play an adversarial min–max game with respect to the Nuclear-norm Wasserstein discrepancy $L_{nwd}$: the classifier *C*, which is reused as an implicit discriminator, maximizes $L_{nwd}$, whereas the feature extractor *G* minimizes it to reduce the discrepancy between the source- and target-domain feature distributions. Following the original DALN formulation, the adversarial optimization is implemented using a gradient reversal layer (GRL), which enables the feature extractor and classifier to be updated simultaneously in a single backpropagation step, rather than through explicit alternating optimization. The classification and domain adaptation objectives are balanced by the trade-off parameter *λ*, which is gradually increased from 0 to 1 during training according to the following schedule:

(9) $$\lambda =\frac{2}{1+e^{-10p}}-1$$

(10) $$p=\frac{\mathrm{current}\;\mathrm{iteration}}{\mathrm{total}\;\mathrm{iterations}}$$

where *p* denotes the normalized training progress, which increases linearly from 0 to 1 throughout the training process. This scheduling strategy gradually increases the contribution of the domain adaptation objective, allowing the network to first learn discriminative source-domain features before progressively emphasizing domain alignment.

**Figure 3:**
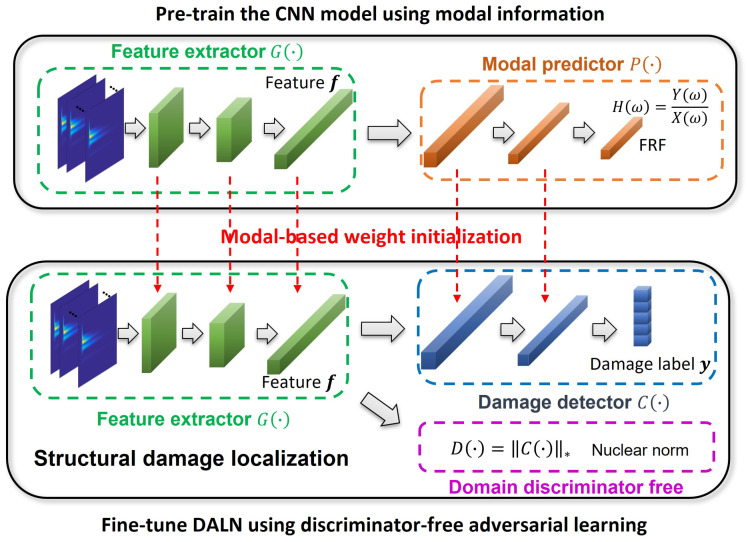
A schematic diagram of physics-informed domain adaptation (PhyDA).

Conventional adversarial domain adaptation methods require unlabeled target-domain data during the adversarial learning process. However, in SHM, it is generally infeasible to collect data representing various damage scenarios from the actual structure, even without corresponding labels. To improve the generalization performance of DALN for structural damage localization without requiring damage-state data from the target domain, a physics-informed machine learning strategy is incorporated into DALN, resulting in the proposed physics-informed domain adaptation (PhyDA) approach. A CNN consisting of a feature extractor G(·) and a modal predictor P(·) is trained using CWTs as inputs to predict the structural modal information represented by the frequency response functions (FRFs). The feature extractor and modal predictor of the CNN share the same architectures as the feature extractor and damage detector of DALN, respectively, except for the output layer. The FRF describes the dynamic relationship between the excitation force and the structural response in the frequency domain, and is defined as

(11) $$H\left(\omega \right)=\frac{X(\omega )}{F(\omega )},$$

where H(ω) denotes the FRF, X(ω) is the Fourier transform of the structural response, and F(ω) is the Fourier transform of the excitation force. FRFs encapsulate the essential modal characteristics of a structure. In particular, natural frequencies correspond to the resonance peak frequencies of the FRFs, whereas mode shapes can be extracted from the amplitude and phase information contained in the FRFs [82]. To incorporate physics-informed constraints into DALN, a modal-based weight initialization strategy is employed. In this strategy, the weights of a CNN pretrained to learn modal information from structural responses are used to initialize the feature extractor and damage detector of DALN (Figure 3), thereby embedding physically meaningful prior knowledge into the learning process. The modal-based weight initialization process can be expressed as

(12) $$W_{G}^{\mathrm{DALN},(0)}=W_{G}^{\mathrm{CNN}},\;W_{C}^{\mathrm{DALN},(0)}=W_{P}^{\mathrm{CNN}},$$

where $W_{G}^{\mathrm{DALN},(0)}$ and $W_{C}^{\mathrm{DALN},(0)}$ denote the initial weights of the feature extractor G(·) and damage detector C(·) in DALN, respectively. $W_{G}^{\mathrm{CNN}}$ and $W_{P}^{\mathrm{CNN}}$ denote the weights of the feature extractor G(·) and modal predictor P(·) learned by the CNN during the modal-information prediction task, respectively. The weights of all shared convolutional layers in the DALN, including Conv1–Conv3 in the feature extractor and Conv1–Conv2 in the damage detector, are initialized using the pretrained weights of the corresponding layers in the CNN feature extractor and modal predictor. Dimensional compatibility is ensured because these corresponding layers have identical architectures and parameter dimensions. The task-specific fully connected output layer is newly initialized because its output dimension differs between modal prediction and damage severity classification. The physically initialized DALN is then fine-tuned through discriminator-free adversarial learning to perform structural damage localization on the higher-fidelity FEM.

Physics-informed machine learning approaches can be broadly categorized into several groups, including physics-informed loss functions, physics-informed initialization, physics-informed network architectures, residual modeling, and hybrid physics–machine learning models [74]. The proposed method belongs to the physics-informed initialization category, where physical knowledge is incorporated during the initialization stage rather than being enforced explicitly through governing equations or physics-based loss functions during network optimization. Randomly initialized neural networks may exhibit slow convergence or converge to suboptimal solutions, particularly in deep architectures. By contrast, introducing prior physical knowledge during initialization can improve training efficiency and guide the network toward more meaningful feature representations [83].

In the proposed framework, a neural network is first trained to predict structural modal information represented by FRFs. Since FRFs capture the dynamic characteristics of a structure, including its natural frequencies and mode shapes, the resulting network learns feature representations associated with structural dynamics. The learned weights are subsequently transferred to initialize the DALN. Unlike conventional transfer learning, which generally relies on pretraining using generic source-domain tasks, the proposed strategy initializes the network using features learned from physically meaningful modal information. This physics-informed initialization provides a more informative starting point for domain adaptation by embedding structural dynamic knowledge into the shared convolutional layers of the feature extractor and damage detector prior to adversarial adaptation.

The proposed PhyDA approach enables the neural network to learn vibration signatures from the reduced-order FEM that are consistent with those of the higher-fidelity FEM, thereby reducing the domain gap between the source and target domains. Consequently, the approach can perform damage localization without requiring damage-state data from the higher-fidelity FEM during training. Moreover, to further evaluate the generalization capability of the proposed approach, the damage scenarios simulated in the reduced-order FEM for training are intentionally designed to be distinct from those in the higher-fidelity FEM. In our previous study, targeted excitation, achieved by applying impact forces at locations near the damage site, was shown to enhance damage identifiability and improve damage localization performance [80]. In practical applications, this strategy can be implemented by systematically applying impact excitations at potential damage locations and retaining only the predictions for which the identified damage location coincides with the corresponding impact location. Therefore, targeted excitation is adopted in this study.

### 2.4. Structural Damage Severity Classification

Building on the HierPhyDA framework for structural damage detection and localization [80], in which anomaly detection first determines whether a structure is damaged and PhyDA is subsequently used for damage localization, the proposed framework further extends the methodology to structural damage severity classification, thereby integrating damage detection, localization, and severity classification into a unified pipeline. Structural damage severity classification adopts the same PhyDA architecture employed for damage localization, consisting of the DALN and CNN architectures summarized in Table 2, with a dedicated PhyDA model trained for each potential damage location. To train each PhyDA model, the same modal-based weight initialization and deep adversarial domain adaptation strategies described in Section 2.3 are employed. The only differences are that the target labels represent damage severities rather than damage locations, and each PhyDA model is trained exclusively using the damage cases associated with a single damage location. During inference, the PhyDA model corresponding to the identified damage location is selected to estimate the damage severity, as illustrated in Figure 1. As summarized in Table 3, the primary contribution of the present study is the integration of the previously developed and validated damage detection and localization modules with the proposed damage location-wise severity classification module into a unified end-to-end SHM framework, thereby providing a comprehensive solution for structural damage identification.

It should be noted that structural damage severity classification is generally more complex than damage detection and damage localization. The proposed integrated hierarchical physics-informed domain adaptation (I-HierPhyDA) framework addresses this challenge by performing damage detection, localization, and severity classification hierarchically, thereby decomposing a complex damage identification problem into a series of more manageable tasks. Furthermore, location-specific damage severity classification is motivated by the fact that damage at different locations can have different effects on structural dynamics. For instance, one-brace removal in lower stories may induce greater changes in the dynamic behavior of a structure than the same level of damage in higher stories, since lower stories support larger loads and contribute more significantly to the overall structural stiffness and stability.

Due to the increased complexity of structural damage severity classification, unlabeled damaged-state data from the target domain (i.e., the higher-fidelity FEM) are incorporated into the adversarial domain adaptation process. This differs from the damage localization stage, which requires only healthy-state data from the target domain. Unsupervised domain adaptation methods are generally classified as transductive transfer learning because they require unlabeled target-domain data during training to align the source and target feature distributions [84]. The proposed damage localization stage overcomes the need for unlabeled damaged-state target-domain data, whereas the damage severity classification stage remains a conventional transductive unsupervised domain adaptation process. The adaptation process is required only when transferring the model to a new target structure. Once adapted, the same model can be applied to different damage states of that target structure without repeating the adaptation process, provided that its underlying structural dynamics remain sufficiently consistent. If significant changes in structural dynamics occur due to factors such as environmental or operational variations, structural modifications, or changes in boundary conditions, the adaptation process may need to be repeated. In practice, unlabeled damaged-state data can be collected through long-term structural health monitoring, during which damage may naturally develop over the service life of the structure. The unsupervised damage detection technique introduced in Section 2.2 can then be used to distinguish healthy-state data from damaged-state data without requiring labeled damage information.

It should be noted that the damage localization and damage severity classification stages follow different protocols. For damage localization, only healthy-state target-domain data are used during domain adaptation, while damaged-state target-domain data are reserved exclusively for testing. Therefore, no damaged target-domain samples are involved in the adaptation process. In contrast, the damage severity classification stage follows the conventional transductive unsupervised domain adaptation protocol, in which unlabeled target-domain samples are available during adaptation for feature distribution alignment. Their labels remain completely hidden throughout the adaptation process and are revealed only for the final performance evaluation. Accordingly, the reported damage severity classification results follow the standard evaluation protocol adopted in transductive unsupervised domain adaptation [84].

### 2.5. Baseline Approaches

In this study, the proposed I-HierPhyDA framework is compared with several baseline and ablation methods, with particular emphasis on structural damage severity classification. Comprehensive assessments of structural damage detection using unsupervised deep autoencoders and structural damage localization using hierarchical physics-informed domain adaptation have been reported in our previous studies [57,80]. Table 4 summarizes the structural damage severity classification approaches evaluated in this study. As a baseline method, the supervised convolutional neural network (SCNN) is trained on source-domain data generated by the reduced-order FEM and directly evaluated on target-domain data generated by the higher-fidelity FEM without domain adaptation. DALN employs domain adaptation to reduce the distribution discrepancy between the source and target domains. Hierarchical SCNN (HierSCNN) incorporates hierarchical damage identification by first performing structural damage detection and then classifying the damage severity if damage is detected. Hierarchical domain adaptation (HierDA) combines hierarchical damage identification and domain adaptation. Physics-informed domain adaptation (PhyDA) integrates physics-informed machine learning with domain adaptation. Hierarchical physics-informed domain adaptation (HierPhyDA) combines hierarchical damage identification, physics-informed machine learning, and domain adaptation. Compared with HierPhyDA, the proposed I-HierPhyDA framework integrates structural damage detection, damage localization, and damage severity classification into a unified hierarchical framework.

A well-established transmissibility-based structural damage detection and quantification approach [85] is also adopted as a baseline method for comparison with the proposed I-HierPhyDA framework. For a single excitation, the transmissibility between sensor locations *r* and *s* can be equivalently expressed as

(13) $$t_{rs}\left(\omega \right)=\frac{H_{rj}\left(\omega \right)}{H_{sj}\left(\omega \right)}=\frac{X_{r}\left(\omega \right)}{X_{s}\left(\omega \right)},$$

where $H_{rj}\left(\omega \right)$ and $H_{sj}\left(\omega \right)$ are the FRFs from excitation location *j* to response locations *r* and *s*, respectively. Structural damage is identified by comparing the transmissibilities of the healthy reference and unknown structural states using the Transmissibility Damage Indicator (TDI), defined as

(14) $$\mathrm{TDI}=\frac{1}{N_{\omega }}\sum_{\omega }\frac{{\left|\sum_{r=1}^{M-1}\sum_{q=1}^{N_{h}}t_{rs}^{\left(u\right)}{\left(t_{rs}^{\left(h,q\right)}\right)}^{*}\right|}^{2}}{\left(\sum_{r=1}^{M-1}\sum_{q=1}^{N_{h}}t_{rs}^{\left(u\right)}{\left(t_{rs}^{\left(u\right)}\right)}^{*}\right)\left(\sum_{r=1}^{M-1}\sum_{q=1}^{N_{h}}t_{rs}^{\left(h,q\right)}{\left(t_{rs}^{\left(h,q\right)}\right)}^{*}\right)},$$

where $t_{rs}^{\left(u\right)}$ denotes the transmissibility of the unknown structural state, and $t_{rs}^{\left(h,q\right)}$ denotes the transmissibility of the *q*th healthy reference sample obtained under the same excitation location as the unknown sample. $N_{h}$ is the number of healthy reference samples corresponding to the same excitation location, *M* is the number of measurement locations, $N_{\omega }$ is the number of frequency samples, and ${\left(\cdot \right)}^{*}$ denotes the complex conjugate. Transmissibilities are calculated between adjacent sensor pairs, i.e., *s* = *r* + 1. For each unknown sample, only healthy reference samples obtained under the same excitation location are used to calculate the TDI. A lower TDI indicates a larger deviation from the healthy state, corresponding to more severe structural damage. This approach performs structural damage detection and quantification directly from measured vibration responses without requiring finite element modeling or modal identification.

## 3. ASCE Benchmark Models

**Figure 4:**
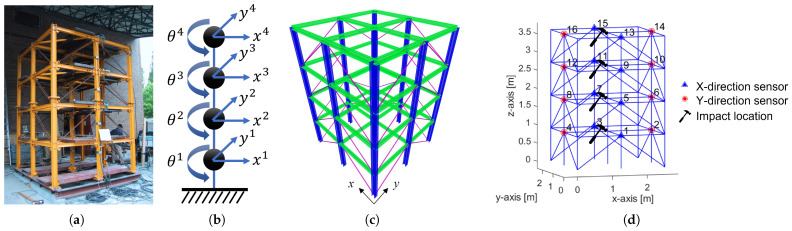
ASCE benchmark structure: (**a**) Photograph of the experimental benchmark structure, shown for illustration only [87]. (**b**) Source structure represented by the 12-DOF reduced-order FEM used to generate the source-domain data. (**c**) Target structure represented by the 120-DOF high-fidelity FEM used to generate the target-domain data. (**d**) Accelerometer and impact locations in the FEMs. Figures (**b**–**d**) are adapted from [80].

In this study, the ASCE benchmark structure is used to evaluate the proposed I-HierPhyDA framework for structural damage detection, localization, and severity classification. The benchmark structure consists of a quarter-scale, four-story steel frame with a two-bay configuration in both horizontal directions (Figure 4a). The structure spans 2.5 m × 2.5 m in plan and has an overall height of 3.6 m. Eight exterior diagonal braces are installed at each story and can be selectively removed to represent different damage conditions. Two FEMs were developed by Johnson et al. [86] to represent the dynamic behavior of the benchmark structure. The first is a simplified 12-DOF shear-building model that permits two horizontal translational DOFs and one rotational DOF at each floor (Figure 4b). The second is a higher-fidelity 120-DOF model that captures additional out-of-plane DOFs, including vertical translation and floor pitching and rolling motions (Figure 4c). The 12-DOF model serves as the source structure (i.e., the reduced-order FEM), whereas the 120-DOF model serves as the target structure (i.e., the higher-fidelity FEM). To simulate the discrepancy between the source and target structures, the 120-DOF model incorporates 5% structural uncertainty and 10% measurement noise [80]. The structural uncertainty is modeled by independently varying Young’s modulus *E*, shear modulus *G*, and density *ρ* according to

(15) $$E=\alpha E_{0},\;G=\beta G_{0},\;\rho =\gamma \rho_{0},$$

where $E_{0}$, $G_{0}$, and $\rho_{0}$ denote the nominal values of the corresponding material properties. The scaling factors *α*, *β*, and *γ* are independently sampled from a uniform distribution over the interval $\left(1-l_{\mathrm{su}}/2,\;1+l_{\mathrm{su}}/2\right)$, where $l_{\mathrm{su}}$ represents the prescribed structural uncertainty level. Measurement noise is introduced using independent Gaussian pulse processes. The root mean square (RMS) of the noise is set to 10% of the maximum RMS among the simulated acceleration responses [86].

Both FEMs are instrumented with 16 uniaxial accelerometers to record the acceleration responses of the structure, with eight sensors aligned in the X-direction and eight in the Y-direction. The structure is excited by an impact force applied at the center of each floor at a 45° angle to the X-axis, as shown in Figure 4d. A sampling frequency of 1000 Hz and a response duration of 4 s are used to generate the simulated acceleration responses for both the reduced-order FEM and the high-fidelity FEM. The acceleration responses are transformed into time–frequency representations using the CWT with the Morse wavelet, resulting in CWT images of size 88 × 104. The corresponding FRFs are discretized into vectors of size 1 × 420. The dataset generation, model training, and testing are performed on a desktop computer equipped with an NVIDIA GeForce RTX 5080 GPU and an Intel Core Ultra 9 285K CPU.

To systematically assess the performance of the proposed I-HierPhyDA framework, a comprehensive set of damage cases was generated for both the 12-DOF and 120-DOF models. Damage was simulated by removing one, two, three, or four braces from a single story. Consequently, 4×81=32, 4×82=112, 4×83=224, and 4×84=280 distinct damage cases were generated for the one-, two-, three-, and four-brace removal scenarios, respectively. For each damage case, five evenly spaced impact force amplitudes ranging from 9000 to 11,000 N were applied at each floor. As a result, a total of 5 × 32 = 160, 5 × 112 = 560, 5 × 224 = 1120, and 5 × 280 = 1400 response cases were generated for the one-, two-, three-, and four-brace removal scenarios, respectively. To ensure a balanced dataset across the healthy and damaged conditions, 160 cases were randomly sampled for each damage category, including 40 cases from each of the one-, two-, three-, and four-brace removal scenarios. Furthermore, to evaluate the generalization capability of the proposed framework, the damage cases in the source structure (i.e., the 12-DOF model) and the target structure (i.e., the 120-DOF model) were designed to be mutually exclusive. Specifically, the damaged braces in the source domain differ from those in the target domain, resulting in different structural dynamic signatures. Consequently, the framework was trained on source-domain damage cases and evaluated on previously unseen target-domain damage cases. During dataset generation, targeted excitation is employed such that the excitation location is consistent with the damage location. Detailed dataset information is provided in Table 5. To train the CAE for structural damage detection, 640 healthy cases are generated using the 120-DOF model with force amplitudes different from those used for the 160 healthy cases reserved for testing.

## 4. Results and Discussion

### 4.1. Bayesian Model Updating

**Figure 5:**
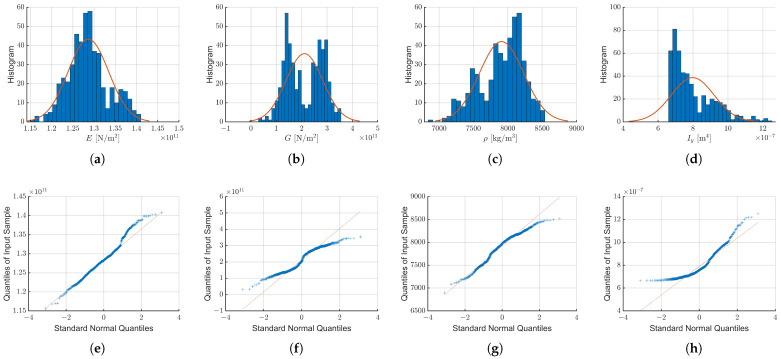
Histograms (blue bars) and fitted normal distributions (red curves) of the updated model parameters of the 12-DOF model: (**a**) Young’s modulus *E*. (**b**) Shear modulus *G*. (**c**) Density *ρ*. (**d**) Moment of inertia $I_{y}$. (**e**–**h**) Quantile–quantile (Q–Q) plots for *E*, *G*, *ρ*, and $I_{y}$, where the blue points represent the sample quantiles and the red dashed lines represent the theoretical normal reference lines, demonstrating the approximate agreement of the data with the fitted normal distributions.

**Figure 6:**
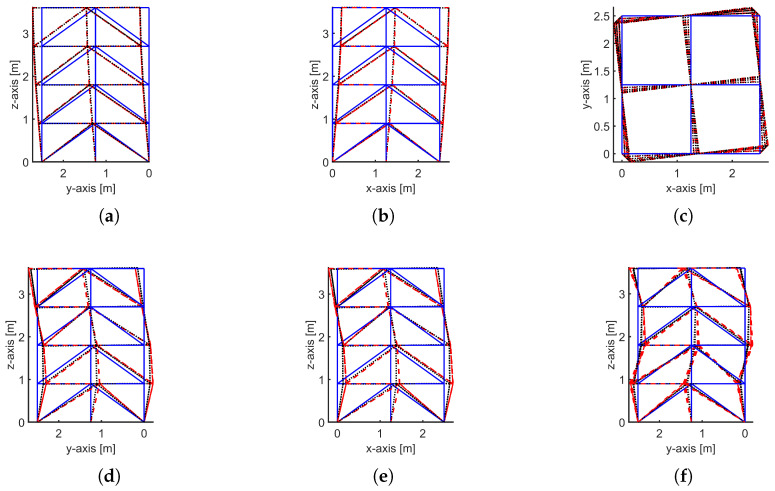
Mode shape comparison between the updated 12-DOF model and the 120-DOF model: (**a**) 1st mode. (**b**) 2nd mode. (**c**) 3rd mode. (**d**) 4th mode. (**e**) 5th mode. (**f**) 6th mode. The blue solid line represents the undeformed shape, the red dashed line represents the mode shapes of the updated 12-DOF model, and the black dotted line represents the mode shapes of the 120-DOF model.

**Figure 7:**
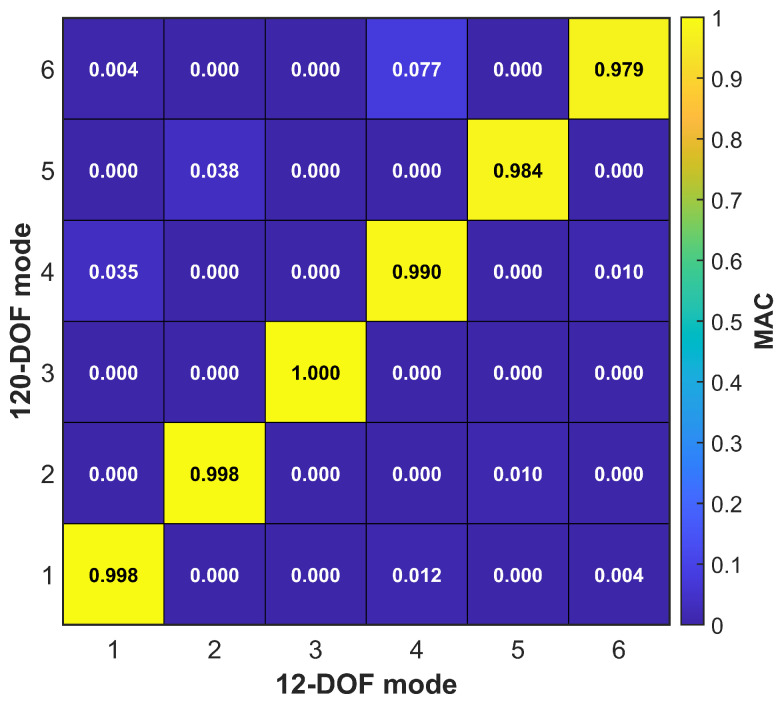
MAC matrix between the updated 12-DOF model and the 120-DOF model.

To mitigate negative transfer in domain adaptation, where knowledge transferred from the source domain adversely affects performance in the target domain, the 12-DOF model is updated using the TMCMC approach [79] to better match the healthy-state dynamics of the 120-DOF model. Specifically, the model parameters of the 12-DOF model, including Young’s modulus *E*, shear modulus *G*, density *ρ*, and moment of inertia in the strong axis $I_{y}$, are updated to match the natural frequencies and mode shapes of the 120-DOF model in the healthy state. In the TMCMC procedure (Appendix A), five transitional stages are employed with posterior powers of $\rho_{1}=0.05$, $\rho_{2}=0.1$, $\rho_{3}=0.2$, $\rho_{4}=0.5$, and $\rho_{5}=1$, starting from the prior distribution ($\rho_{0}=0$). A total of 500 samples are generated at each stage. The histogram with a normal distribution fit of each updated model parameter is demonstrated in Figure 5. The mean of the fitted normal distribution is utilized for each updated model parameter. The frequency error $f_{err}$ between source and target structures is calculated as follows:

(16) $$f_{err}=\frac{f_{s}-f_{t}}{f_{t}}\times 100\%,$$

where $f_{s}$ is the natural frequency of the source structure, while $f_{t}$ is the natural frequency of the target structure. The modal assurance criterion (MAC) is employed to assess the degree of collinearity between the mode shapes of the source structure and those of the target structure. The MAC matrix can be calculated as follows:

(17) $$MAC_{ij}=\frac{{\left[\Phi_{s,i}^{T}\Phi_{t,j}\right]}^{2}}{\left[\Phi_{s,i}^{T}\Phi_{s,i}\right]\left[\Phi_{t,j}^{T}\Phi_{t,j}\right]},$$

where $\Phi_{s,i}$ denotes the mode shape vector of the $i^{th}$ mode of the source structure, while $\Phi_{t,j}$ denotes the mode shape vector of the $j^{th}$ mode of the target structure. When the mode shapes of the source structure closely resemble those of the target structure, the diagonal components of the MAC matrix tend to approach 1, while the off-diagonal components tend to approach 0. Table 6 and Figure 6 and Figure 7 compare the natural frequencies and mode shapes between the updated 12-DOF model and the 120-DOF model, demonstrating a strong correlation between the two models. However, inevitable discrepancies remain due to model simplifications and uncertainties in material properties and boundary conditions. Consequently, physics-informed domain adaptation is introduced to further bridge the gap between the 12-DOF model and the 120-DOF model by learning domain-invariant features that capture the common vibration characteristics shared by both source and target structures.

### 4.2. Structural Damage Detection and Localization

**Figure 8:**
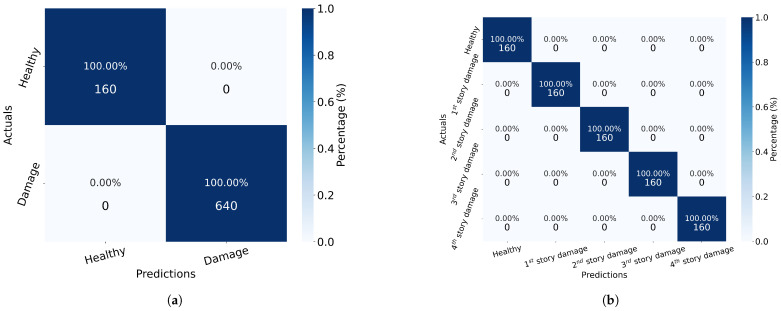
Confusion matrices for (**a**) structural damage detection and (**b**) structural damage localization, both achieving an accuracy of 100.00%.

The preliminary step in the proposed I-HierPhyDA framework is to detect structural anomalies using unsupervised CAE. The CAE for structural damage detection is trained using the Adam optimizer with a learning rate of 0.005, a batch size of 10, and 250 training epochs. The training and inference times of the CAE are 18.5 min and $5.4\times 10^{-4}$ s, respectively. According to the previous study [57], the threshold *τ* is selected as the 95th percentile of the MSE distribution fitted to the training data in the healthy state. Additionally, the threshold *m* for sensor fusion is selected as 8 out of 16 sensors, corresponding to a majority vote in the decision-making process. Once the damage is detected within the structural system, the next phase is to localize the damage using PhyDA [80]. The PhyDA modules for structural damage localization and location-specific damage severity classification are pretrained using stochastic gradient descent (SGD) with a learning rate of 0.1, a batch size of 10, and 10 training epochs. They are then fine-tuned using SGD with learning rates of 0.001 and 0.0001 for the feature extractor and damage detector, respectively, with a batch size of 10 for 10 training epochs. The training and inference times of the PhyDA module are 60.3 s and $1.5\times 10^{-4}$ s, respectively. Structural damage localization requires training and inference of a single PhyDA module, whereas structural damage severity classification requires training and inference of four location-specific PhyDA modules. The confusion matrices for structural damage detection and localization are shown in Figure 8. The proposed approaches achieve 100% accuracy for both structural damage detection and localization. Structural damage detection and localization have been extensively investigated in our previous studies under various challenging conditions, including different levels of structural uncertainty and measurement noise, limited sensor layouts, and limited training samples. Furthermore, both the structural damage detection and localization modules have been experimentally validated using a full-scale field structure in our previous publications [57,80]. Therefore, these comprehensive robustness evaluations are not repeated in the present study, whose primary objective is to integrate the previously developed and validated damage detection and localization modules with the proposed location-specific damage severity classification module into a unified end-to-end structural health monitoring framework.

### 4.3. Structural Damage Severity Classification

**Figure 9:**
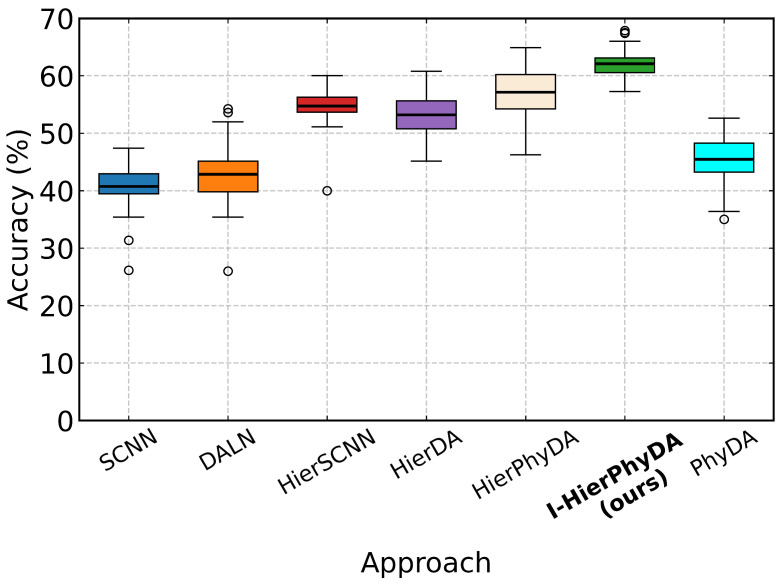
Comparison of structural damage severity classification accuracies achieved by different approaches over 30 independent training runs. In each boxplot, the center line represents the median accuracy, the box spans the first (Q1) and third (Q3) quartiles, the whiskers extend to the most extreme values within 1.5× the interquartile range (IQR), and the circles denote outliers. The corresponding numerical summary statistics are reported in Table 7.

Following structural damage detection using unsupervised CAE and structural damage localization through PhyDA, the proposed I-HierPhyDA framework proceeds to the final phase, where the severity of damage at the identified locations is classified using dedicated location-specific PhyDA models. To account for the variability introduced by random network initialization, each approach listed in Table 4 is independently trained and tested 30 times. Figure 9 presents box plots comparing the structural damage severity classification performance of the different approaches. The results demonstrate that DALN achieves improved damage severity classification performance compared with SCNN by bridging the gap between the source and target structures through adversarial domain adaptation. Moreover, the approaches incorporating the hierarchical damage identification strategy (i.e., HierSCNN, HierDA, HierPhyDA, and I-HierPhyDA), which first detect structural anomalies and subsequently classify damage severity only when the structure is identified as damaged, achieve superior performance compared with non-hierarchical damage identification approaches (i.e., SCNN, DALN, and PhyDA). Additionally, by integrating hierarchical damage identification, physics-informed machine learning, and domain adaptation, HierPhyDA and I-HierPhyDA rank among the top-performing approaches, achieving the second-highest and highest damage severity classification accuracies, respectively.

As shown in Figure 9 and Table 7, compared with HierPhyDA, the proposed I-HierPhyDA framework further integrates structural damage detection, localization, and severity classification into a unified hierarchical framework, resulting in the highest mean accuracy of 62.10%, the highest maximum accuracy of 67.88%, and the lowest standard deviation of 2.70% among all the approaches considered. The modal-based weight initialization constrains the damage diagnosis model to learn vibration signatures shared by the source and target structures, thereby effectively reducing the variability in estimation performance across 30 independent train/test runs, as evidenced by the lowest standard deviation among the compared approaches. To statistically evaluate the performance of the different approaches, the mean accuracy and standard deviation are summarized in Table 7. Additionally, Wilcoxon rank-sum tests [88] are conducted to assess the statistical significance of the performance improvements achieved by I-HierPhyDA over the baseline approaches. As shown in Table 7, the *p*-values are obtained by comparing the performance of I-HierPhyDA with that of each baseline approach. All *p*-values are less than 0.05, except for the self-comparison, indicating that the performance improvements achieved by I-HierPhyDA are statistically significant. Figure 13 presents the confusion matrices for structural damage severity classification obtained using the best-trained HierPhyDA and I-HierPhyDA models, achieving accuracies of 64.88% and 73.88%, respectively. It should be noted that the reported 73.88% accuracy does not correspond to the performance of a single I-HierPhyDA model realization. Instead, it is obtained by retrospectively integrating the best-performing PhyDA model from 30 independent runs for each story. Therefore, this result represents an upper-bound (oracle) performance of the proposed I-HierPhyDA framework rather than its expected operational performance.

**Figure 10:**
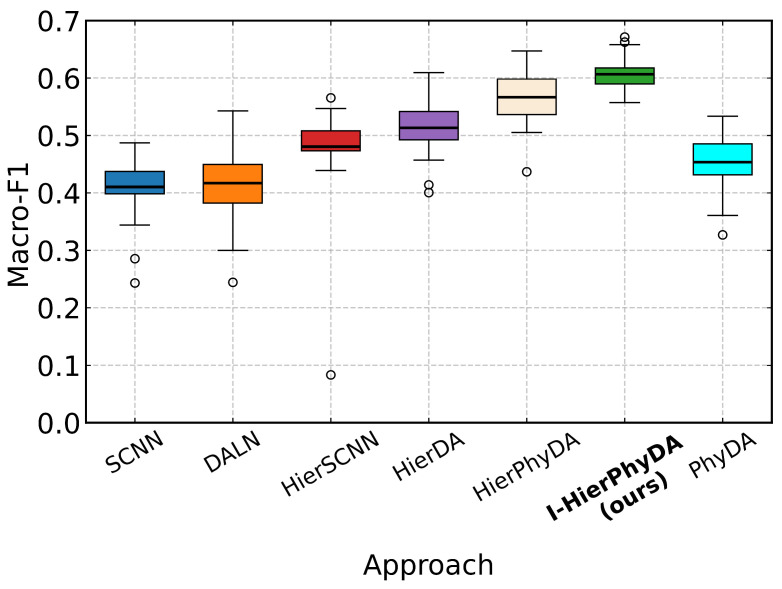
Comparison of structural damage severity classification macro-F1 scores achieved by different approaches.

**Figure 11:**
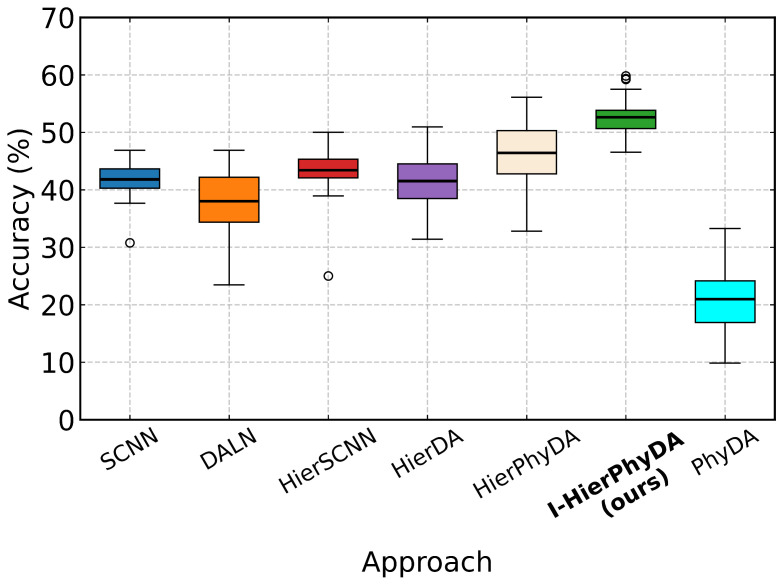
Comparison of damage severity classification accuracies achieved by different approaches using damaged samples only.

**Figure 12:**
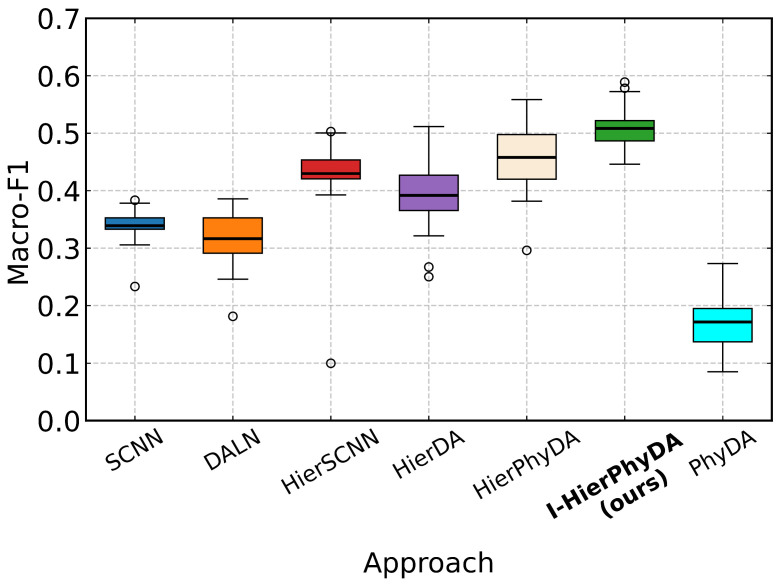
Comparison of structural damage severity classification macro-F1 scores achieved by different approaches using damaged samples only.

In addition to classification accuracy, the Macro-F1 score is reported to provide a more comprehensive evaluation of multiclass damage severity classification performance. Unlike accuracy, which measures the overall proportion of correctly classified samples, the Macro-F1 score accounts for both precision and recall for each damage class while assigning equal importance to all classes. Consequently, it provides a more balanced assessment of the classifier’s ability to distinguish among different damage scenarios, particularly when some classes are more challenging to identify than others. As shown in Figure 10 and Table 8, the proposed I-HierPhyDA framework achieves the highest mean and maximum Macro-F1 scores, together with the lowest standard deviation, demonstrating both superior performance and greater consistency across 30 independent runs. Furthermore, the Wilcoxon rank-sum test results indicate that the improvements achieved by I-HierPhyDA over the baseline approaches are statistically significant in terms of the Macro-F1 score. Figure 9 and Figure 10, together with Table 7 and Table 8, present the mean and standard deviation of the accuracy and Macro-F1 score, along with the corresponding *p*-values, for the end-to-end structural-condition assessment of both healthy and damaged structural states. The corresponding results for damage severity classification conditioned on damaged samples only are presented in Figure 11 and Figure 12, together with Table 9 and Table 10.

It is noteworthy that both HierPhyDA and I-HierPhyDA achieve higher damage severity classification accuracies for one-brace and four-brace removal scenarios, whereas lower accuracies are observed for two-brace and three-brace removal scenarios. This suggests that the models can more readily distinguish the least and most severe damage states, while intermediate damage states are more difficult to differentiate due to their similar structural response characteristics. As shown in Figure 15b,d,f, the mean modal frequencies vary gradually with increasing damage severity, resulting in relatively small differences between adjacent damage-severity levels. Consequently, intermediate damage states are frequently misclassified as adjacent damage categories. For example, two-brace removal cases may be classified as one-brace or three-brace removal, whereas three-brace removal cases may be classified as two-brace or four-brace removal. These results indicate that the majority of misclassifications occur between adjacent damage-severity levels, leading to modest underestimation or overestimation of damage severity rather than fundamentally incorrect damage assessments.

**Figure 13:**
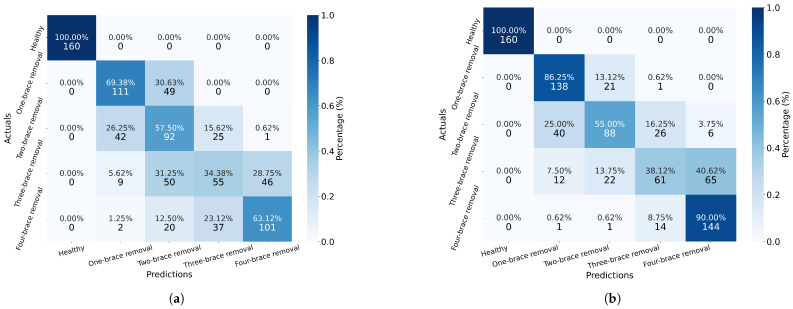
Confusion matrices for structural damage severity classification: (**a**) HierPhyDA (accuracy = 64.88%) and (**b**) I-HierPhyDA (accuracy = 73.88%). The I-HierPhyDA results are obtained by retrospectively integrating the predictions of the best-trained PhyDA model for each story, rather than from a single end-to-end trained model.

**Figure 14:**
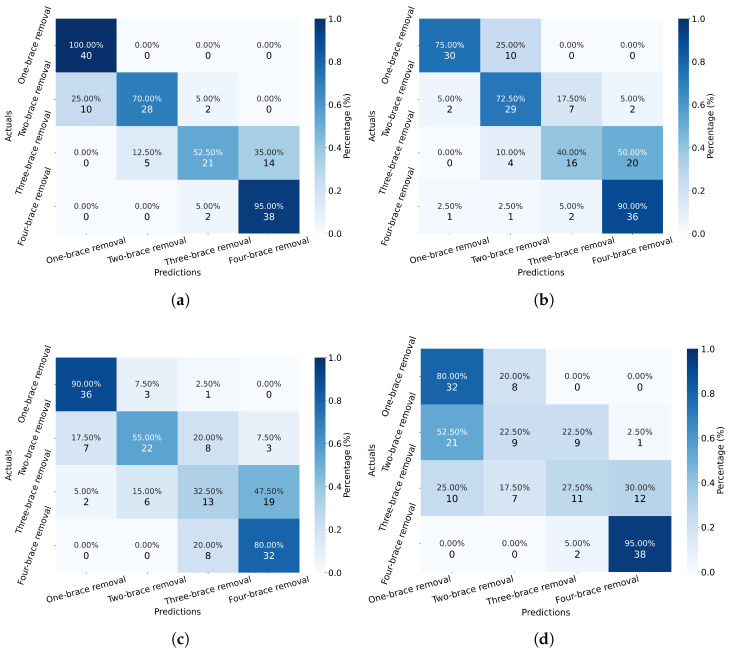
Confusion matrices for structural damage severity classification using the best-trained PhyDA models for each story in the I-HierPhyDA framework: (**a**) 1st-story damage, (**b**) 2nd-story damage, (**c**) 3rd-story damage, and (**d**) 4th-story damage, achieving accuracies of 79.38%, 69.38%, 64.38%, and 56.25%, respectively.

The confusion matrix of the best-trained I-HierPhyDA model shown in Figure 13b can be further decomposed into individual confusion matrices corresponding to each story, as presented in Figure 14. It can be observed that the accuracy of damage severity classification decreases as the damage location moves to higher stories, achieving accuracies of 79.38%, 69.38%, 64.38%, and 56.25% for 1st-, 2nd-, 3rd-, and 4th-story damage, respectively. This can be attributed to the fact that the same number of brace removals may have different effects on structural dynamics depending on the story where the damage occurs. Because lower stories support larger loads and contribute more significantly to the overall structural stiffness and stability, the same level of damage in lower stories produces more pronounced changes in the structural response than in higher stories. As a result, damage in lower stories is more readily identifiable, leading to higher damage severity classification accuracy. To further investigate the influence of damage location on damage severity classification performance, the following analyses are performed using two damage identifiability measures: modal frequency variation-based damage identifiability and weighted generalized stiffness reduction-based damage identifiability.

**Figure 15:**
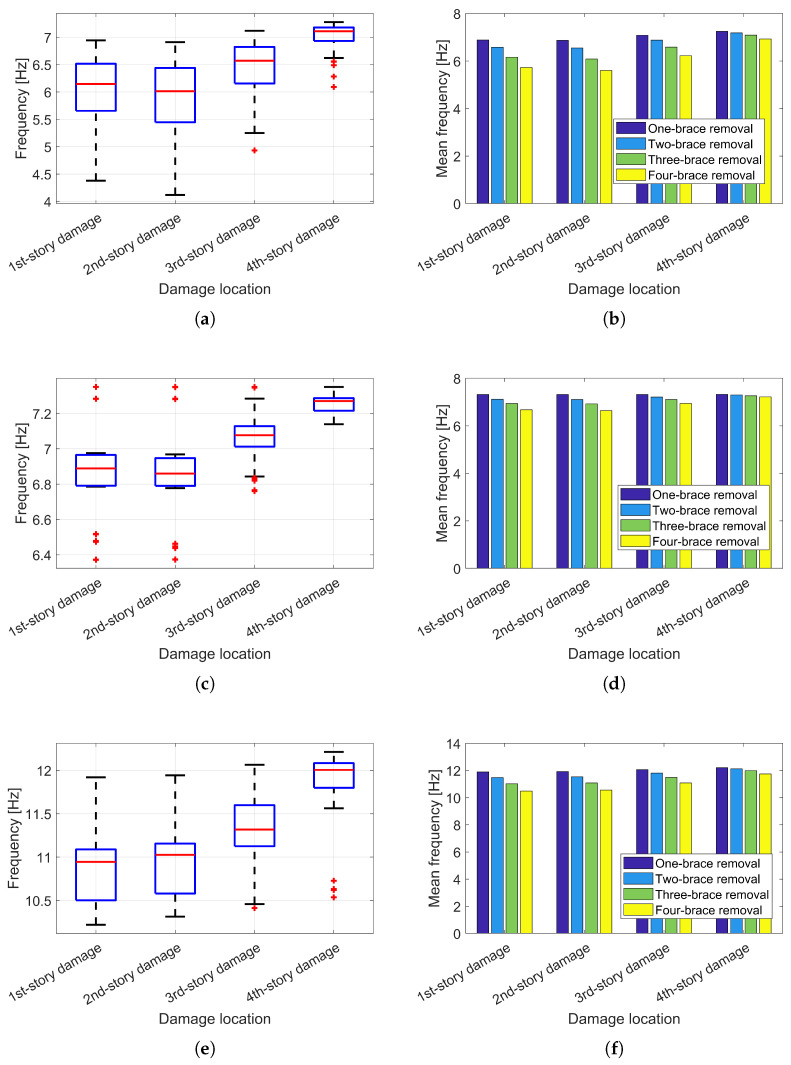
Modal frequency variation-based damage identifiability for the first three modes: (**a**,**c**,**e**) boxplots of the modal frequencies for the 1st, 2nd, and 3rd modes, respectively, for all damage severity levels at each damage location; (**b**,**d**,**f**) corresponding mean modal frequencies for each damage severity at each damage location.

Damage identifiability can be assessed by the variation in modal frequencies under different damage severities. Greater variations in modal frequencies indicate higher damage identifiability and, consequently, facilitate more accurate damage severity classification. The first three vibration modes are considered because they capture the dominant global structural dynamic characteristics, contribute most significantly to the global dynamic response of the structure, and therefore provide the most informative modal information for structural damage identification. As shown in Figure 15, lower-story damage, such as the 1st-story damage, results in larger variations in modal frequencies across one-, two-, three-, and four-brace removal cases. In contrast, higher-story damage, such as the 4th-story damage, produces smaller variations in modal frequencies across different damage severity levels. This is because the lower-story braces carry greater loads than the higher-story braces and contribute more to the overall structural stiffness. Consequently, damage in the lower stories has a greater influence on the structural dynamics, resulting in larger modal frequency variations and, therefore, higher damage identifiability. This explains why, as shown in Figure 14, the PhyDA model achieves the highest damage severity classification accuracy for the 1st-story damage.

**Figure 16:**
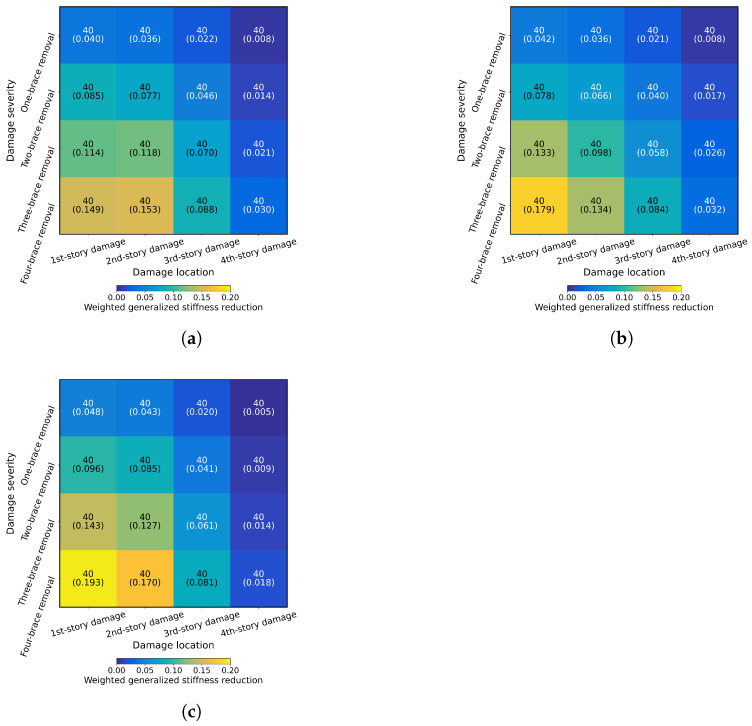
Weighted generalized stiffness reduction-based damage identifiability: (**a**) X-direction excitation, (**b**) Y-direction excitation, and (**c**) torsional excitation. In each cell, the top number indicates the total number of damage cases, whereas the bottom number represents the mean weighted generalized stiffness reduction computed over those cases.

Another metric for evaluating damage identifiability is the weighted generalized stiffness reduction, whose detailed formulation is provided in Appendix B. As shown in Figure 16, the weighted generalized stiffness reduction decreases as the damage location shifts to higher stories. It also decreases as fewer braces are removed, corresponding to lower damage severity. These trends are consistently observed under X-direction, Y-direction, and torsional excitations. These results further confirm that damage in lower stories induces more pronounced changes in the structural dynamics than damage in higher stories, resulting in higher damage identifiability and consequently improved damage severity classification accuracy. The analyses of modal frequency variation-based damage identifiability and weighted generalized stiffness reduction-based damage identifiability collectively demonstrate the significant influence of damage location on damage severity classification performance. These findings further justify the proposed I-HierPhyDA framework, in which structural damage is first detected, then localized, and finally classified into damage severity levels using the location-specific PhyDA model corresponding to the identified damage location.

**Figure 17:**
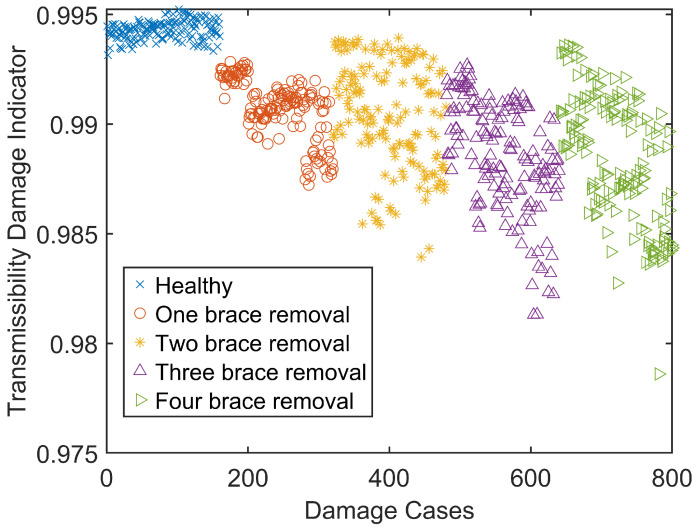
Transmissibility-based structural damage detection and quantification.

The transmissibility-based structural damage detection and quantification approach [85] was evaluated on the same testing dataset by calculating the TDI through comparison of each testing sample with the healthy-state reference dataset used to train the CAE for structural damage detection. As shown in Figure 17, the TDI values are close to 1 for healthy structures and decrease as structural damage is introduced. However, the transmissibility-based approach fails to effectively distinguish different damage severity levels, as the TDI values corresponding to different damage severities exhibit substantial overlap. These results further demonstrate the effectiveness of the proposed approach for structural damage detection and damage severity classification relative to the transmissibility-based baseline.

## 5. Conclusions

In SHM, obtaining labeled damage-state data from actual structures for training data-driven models is often impractical. Although FEMs can generate large amounts of synthetic data under diverse damage scenarios, the domain gap between synthetic and measured data may limit the generalization performance of models trained solely on synthetic data. To address this challenge, this work proposes the I-HierPhyDA framework, which integrates the three key tasks of structural damage identification: damage detection, localization, and severity classification. In this study, the proposed framework is systematically evaluated using the ASCE benchmark reduced-order FEM (source domain) and higher-fidelity FEM (target domain). Based on these numerical studies, the following conclusions can be drawn:

1. The proposed integrated hierarchical damage identification framework sequentially detects structural anomalies, localizes the damage when anomalies are identified, and classifies the damage severity using the corresponding location-specific PhyDA model. The framework achieves accurate and reliable structural damage identification while outperforming its non-integrated and non-hierarchical counterparts.
2. The proposed physics-informed domain adaptation with modal-based weight initialization significantly improves the generalization of damage localization and severity classification models from the source domain (generated by the reduced-order FEM) to the target domain (generated by the higher-fidelity FEM). This is evidenced by its superior damage localization performance without requiring target-domain damage-state data during adaptation and its superior damage severity classification performance using unlabeled target-domain damage-state data during adaptation.
3. The proposed I-HierPhyDA framework achieves 100% accuracy in structural damage detection and localization, as well as the highest mean accuracy and Macro-F1 score and the lowest standard deviation for both metrics in structural damage severity classification among the baseline and ablation methods. Furthermore, analyses of damage identifiability using modal frequency variation and weighted generalized stiffness reduction reveal that damage location significantly influences damage severity classification performance, thereby providing further justification for the proposed integrated hierarchical damage identification framework.
4. The proposed I-HierPhyDA framework has been numerically validated using the ASCE benchmark models under structural uncertainty and measurement noise. Future work will focus on experimentally validating the proposed framework using measured data from laboratory or field structures, as well as evaluating its generalization across different structural types and varying environmental and operational conditions.

## Figures and Tables

**Table 1:** CAE architecture.

Layer	Input Size	Output Size	Output Channels	Kernel Size	Stride	Activation
Conv1	1 × 88 × 104	32 × 44 × 52	32	2 × 2	2 × 2	LeakyReLU
Conv2	32 × 44 × 52	64 × 22 × 26	64	2 × 2	2 × 2	LeakyReLU
Conv3	64 × 22 × 26	128 × 11 × 13	128	2 × 2	2 × 2	LeakyReLU
Conv4	128 × 11 × 13	256 × 5 × 6	256	2 × 2	2 × 2	LeakyReLU
Conv5	256 × 5 × 6	512 × 2 × 3	512	2 × 2	2 × 2	LeakyReLU
Conv6	512 × 2 × 3	1024 × 1 × 1	1024	2 × 2	2 × 2	LeakyReLU
Deconv1	1024 × 1 × 1	512 × 2 × 3	512	2 × 3	2 × 2	LeakyReLU
Deconv2	512 × 2 × 3	256 × 5 × 6	256	3 × 2	2 × 2	LeakyReLU
Deconv3	256 × 5 × 6	128 × 11 × 13	128	3 × 3	2 × 2	LeakyReLU
Deconv4	128 × 11 × 13	64 × 22 × 26	64	2 × 2	2 × 2	LeakyReLU
Deconv5	64 × 22 × 26	32 × 44 × 52	32	2 × 2	2 × 2	LeakyReLU
Deconv6	32 × 44 × 52	1 × 88 × 104	1	2 × 2	2 × 2	LeakyReLU

**Table 2:** Architectures of the feature extractor and damage detector in DALN.

Module	Layer	Input Size	Output Size	Output Channels	Kernel Size	Stride	Activation
Feature extractor	Conv1	16 × 88 × 104	10 × 40 × 48	10	10 × 10	2 × 2	LeakyReLU
	Conv2	10 × 40 × 48	6 × 37 × 45	6	4 × 4	1 × 1	LeakyReLU
	Conv3	6 × 37 × 45	3 × 34 × 42	3	4 × 4	1 × 1	LeakyReLU
Damage detector	Conv1	3 × 34 × 42	2 × 31 × 39	2	4 × 4	1 × 1	LeakyReLU
	Conv2	2 × 31 × 39	1 × 28 × 36	1	4 × 4	1 × 1	LeakyReLU
	Flatten	1 × 28 × 36	1008	—	—	—	—
	FC	1008	4	—	—	—	Linear

**Table 3:** Comparison between the present study and previous studies.

Study	Damage Detection	Damage Localziation	Severity Classification	Main Contrubution
[57]	✓	×	×	Data-driven anomaly detection
[80]	✓	✓	×	Physcis-informed hybrid digital twins
This study	✓	✓	✓	Unified end-to-end SHM framework

**Table 4:** Structural damage severity classification approaches.

Approach	Domain Adaptation	Physics- Informed ML	Hierarchical Damage Identification	
			Damage Detection	Damage Localization
SCNN	×	×	×	×
DALN	✓	×	×	×
HierSCNN	×	×	✓	×
HierDA	✓	×	✓	×
HierPhyDA	✓	✓	✓	×
**I-HierPhyDA (ours)**	✓	✓	✓	✓
PhyDA	✓	✓	×	×

**Table 5:** Dataset information.

Structural Conditions	Source Structure (12-DOF Model)				Target Structure (120-DOF Model)			
	Total Cases	Sampled Cases	Labeled CWTs	FRFs	Total Cases	Sampled Cases	Unlabeled CWTs	FRFs
Healthy	160	160	✓	✓	160	160	✓	✓
1-brace removal	160	160	✓	✓	160	160	✓	×
2-brace removal	560	160	✓	✓	560	160	✓	×
3-brace removal	1120	160	✓	✓	1120	160	✓	×
4-brace removal	1400	160	✓	✓	1400	160	✓	×

Each case contains the data from 16 sensors. To fine-tune the PhyDA module using DALN for damage localization, the labeled CWTs of the damaged cases from the source structure and the unlabeled CWTs of the healthy cases from the target structure are used. The latter are the same as those used to train the CAE for structural damage detection. In contrast, to fine-tune the PhyDA module using DALN for damage severity classification, the labeled CWTs from the damaged cases of the source structure and the unlabeled CWTs from the damaged cases of the target structure are used.

**Table 6:** Natural frequency and mode shape comparison between the updated 12-DOF model and the 120-DOF model.

Mode *i*	12-DOF Model [Hz]	120-DOF Model [Hz]	*f*_err_ [%]	*MAC_ii_*
1st	7.44	7.28	2.16	0.998
2nd	7.65	7.35	4.08	0.998
3rd	11.91	12.26	−2.85	1.000
4th	20.25	20.05	0.95	0.990
5th	20.81	20.39	2.05	0.984
6th	30.64	31.70	−3.36	0.979

**Table 7:** Statistical comparison of structural damage severity classification accuracies achieved by different approaches.

Method	Mean Accuracy [%]	Standard Deviation [%]	Maximum Accuracy [%]	*p*-Value
SCNN	40.75	4.21	47.38	2.87 × 10^−11^
DALN	42.84	5.70	54.25	2.87 × 10^−11^
HierSCNN	54.74	3.44	60.00	1.54 × 10^−10^
HierDA	53.21	3.81	60.75	2.61 × 10^−10^
HierPhyDA	57.15	4.11	64.88	6.07 × 10^−6^
**I-HierPhyDA (Ours)**	**62.10**	**2.70**	**67.88**	1.00
PhyDA	45.45	4.30	52.63	2.87 × 10^−11^

**Table 8:** Statistical comparison of structural damage severity classification Macro-F1 scores achieved by different approaches.

Method	Mean Macro-F1	Standard Deviation	Maximum Macro-F1	*p*-Value
SCNN	0.41	0.05	0.49	2.87 × 10^−11^
DALN	0.42	0.06	0.54	2.87 × 10^−11^
HierSCNN	0.48	0.08	0.57	3.88 × 10^−11^
HierDA	0.51	0.04	0.67	4.40 × 10^−10^
HierPhyDA	0.57	0.05	0.65	4.58 × 10^−4^
**I-HierPhyDA (Ours)**	**0.61**	**0.03**	**0.67**	1.00
PhyDA	0.45	0.05	0.53	2.87 × 10^−11^

**Table 9:** Statistical comparison of structural damage severity classification accuracies achieved by different approaches using damaged samples only.

Method	Mean Accuracy [%]	Standard Deviation [%]	Maximum Accuracy [%]	*p*-Value
SCNN	41.82	**3.20**	46.88	3.18 × 10^−11^
DALN	38.03	5.60	46.88	3.18 × 10^−11^
HierSCNN	43.43	4.30	50.00	1.54 × 10^−10^
HierDA	41.51	4.76	50.93	2.61 × 10^−10^
HierPhyDA	46.44	5.14	56.09	6.07 × 10^−6^
**I-HierPhyDA (Ours)**	**52.62**	3.38	**59.84**	1.00
PhyDA	20.97	5.48	33.28	2.87 × 10^−11^

**Table 10:** Statistical comparison of structural damage severity classification Macro-F1 scores achieved by different approaches using damaged samples only.

Method	Mean Macro-F1	Standard Deviation	Maximum Macro-F1	*p*-Value
SCNN	0.34	**0.03**	0.38	2.87 × 10^−11^
DALN	0.32	0.04	0.39	2.87 × 10^−11^
HierSCNN	0.43	0.07	0.50	1.63 × 10^−8^
HierDA	0.39	0.05	0.51	4.40 × 10^−10^
HierPhyDA	0.46	0.06	0.56	4.58 × 10^−4^
**I-HierPhyDA (Ours)**	**0.51**	0.04	**0.59**	1.00
PhyDA	0.17	0.05	0.27	2.87 × 10^−11^

## Data Availability

Dataset available on request from the authors.

## Abbreviations

The following abbreviations are used in this manuscript:

AE	Autoencoder
ASCE	American Society of Civil Engineers
CAE	Convolutional autoencoder
CNN	Convolutional neural network
CWT	Continuous wavelet transform
DALN	Discriminator-free adversarial learning network
DOF	Degree of freedom
FEM	Finite element model
FRF	Frequency response function
HierPhyDA	Hierarchical physics-informed domain adaptation
I-HierPhyDA	Integrated hierarchical physics-informed domain adaptation
MAC	Modal assurance criterion
MSE	Mean squared error
PhyDA	Physics-informed domain adaptation
SCNN	Supervised convolutional neural network
SHM	Structural health monitoring
TMCMC	Transitional Markov chain Monte Carlo

## Appendix A. TMCMC Model Updating

### Appendix A.1

In Bayesian learning, the posterior distribution p(θ|d,M) of model parameters ***θ*** given response ***d*** and model class M can be estimated from the prior distribution p(θ|M) and likelihood p(d|θ,M). In this study, the model parameters, including Young’s modulus *E*, shear modulus *G*, material density *ρ*, and moment of inertia $I_{y}$, are calibrated to minimize the discrepancy in dynamic characteristics between the FEM and the actual structure. TMCMC estimates a sequence of intermediate posterior distributions $p_{j}\left(\theta \right)$ that gradually transition from the prior distribution to the target posterior distribution [79]:

(A1) $$p_{j}\left(\theta \right)\propto p{\left(d|\theta ,M\right)}^{\rho_{j}}p\left(\theta |M\right),\;j=0,\ldots ,m,\;0=\rho_{0}<\rho_{1}<\cdots <\rho_{m}=1.$$

The basic TMCMC algorithm is as follows:

1. Obtain samples $\theta_{0}=\left[E_{0},G_{0},\rho_{0},I_{y0}\right]$ from the prior distributions $p_{0}\left(\theta \right)$.
2. Repeat the following steps *m* − 1 times:
  (a) Compute the modal parameters of the FEM model using the samples from the previous stage.
  (b) Estimate the Gaussian likelihood $p(d|\theta_{j},M)$ using the modal-parameter errors [89], with the standard deviation set to *σ* = 1.5:
    (A2) $$p\left(d|\theta_{j},M\right)=\frac{1}{\sqrt{2\pi }\sigma }\exp\;\left(-\frac{{\left(e_{1}+e_{2}\right)}^{2}}{2\sigma^{2}}\right)$$
  (c) Compute the plausibility weights:
    (A3) $$w\left(\theta_{j,k}\right)=p{\left(d|\theta_{j,k},M\right)}^{\rho_{j+1}-\rho_{j}}.$$
  (d) Normalize the plausibility weights and use normalized plausibility weights to choose leaders for generating the samples in the next stage.
  (e) For each leader, generate a Metropolis-Hastings (MH) chain with length equal to the number of times a leader was selected in the above step.
  (f) Generate samples for the next stage by sampling the MH chains.

### Appendix A.2

The modal-parameter error consists of two components:

1. Natural frequency error:
  (A4) $$e_{1}=\sum_{k=1}^{N_{m}}\left|f_{FEM,k}-f_{exp,k}\right|,$$
  where $f_{exp,k}$ and $f_{FEM,k}$ are the $k^{\mathrm{th}}$ natural frequencies of the actual structure and FEM model, respectively. $N_{m}$ is the number of modes used in Bayesian model updating.
2. Mode shape error:
  (A5) $$e_{2}=\sum_{i=1}^{N_{m}}\sum_{\begin{array}{c} j=1\\ i\neq j \end{array}}^{N_{m}}MAC_{ij}.$$
  The MAC is computed as
  (A6) $$MAC_{ij}\left(\vartheta \right)=\frac{{\left[\Phi_{FEM,i}^{T}\left(\vartheta \right)\Phi_{exp,j}\left(\vartheta \right)\right]}^{2}}{\left[\Phi_{FEM,i}^{T}\left(\vartheta \right)\Phi_{FEM,i}\left(\vartheta \right)\right]\left[\Phi_{exp,j}^{T}\left(\vartheta \right)\Phi_{exp,j}\left(\vartheta \right)\right]}.$$
  where ϑ is the sensor location parameter, and $\Phi_{i}\left(\vartheta \right)$ represents the ϑ rows of the $i^{\mathrm{th}}$ column vector in the mode shape matrix. MAC is a $N_{m}\times N_{m}$ matrix whose diagonal elements are close to 1 for a perfect match, while off-diagonal elements are close to 0.

## Appendix B. Weighted Generalized Stiffness Reduction

The modal properties of the healthy structure are obtained by solving the generalized eigenvalue problem

(A7) $$K_{h}\phi_{h,i}=\lambda_{h,i}M_{h}\phi_{h,i},$$

where $K_{h}$ and $M_{h}$ are the stiffness and mass matrices of the healthy structure, respectively, $\phi_{h,i}$ is the $i^{\mathrm{th}}$ healthy mode shape, and $\lambda_{h,i}=\omega_{h,i}^{2}$ is the corresponding eigenvalue. In this study, each mode shape is normalized such that its largest absolute component is equal to one. To compare the healthy and damaged structures using a common modal basis, the generalized stiffnesses of both structures are projected onto the healthy mode shapes as

(A8) $$k_{h,i}^{*}=\phi_{h,i}^{T}K_{h}\phi_{h,i},\;k_{d,i}^{*}=\phi_{h,i}^{T}K_{d}\phi_{h,i},$$

where $K_{d}$ is the stiffness matrix of the damaged structure. The generalized stiffness reduction associated with the $i^{\mathrm{th}}$ mode is then defined as

(A9) $$r_{i}=1-\frac{k_{d,i}^{*}}{k_{h,i}^{*}}=1-\frac{\phi_{h,i}^{T}K_{d}\phi_{h,i}}{\phi_{h,i}^{T}K_{h}\phi_{h,i}}.$$

A positive value of $r_{i}$ therefore indicates a reduction in generalized stiffness caused by damage. For an influence vector l, the effective modal mass of the $i^{\mathrm{th}}$ healthy mode is calculated as

(A10) $$m_{i}^{\mathrm{eff}}\left(l\right)=\frac{{\left(\phi_{h,i}^{T}M_{h}l\right)}^{2}}{\phi_{h,i}^{T}M_{h}\phi_{h,i}},$$

where l is the influence vector corresponding to the excitation direction under consideration. Separate influence vectors are used for the horizontal translational, vertical translational, and rotational directions. The effective-modal-mass-weighted generalized stiffness reduction is finally computed as

(A11) $$R\left(l\right)=\frac{\sum_{i=1}^{N_{m}}m_{i}^{\mathrm{eff}}\left(l\right)r_{i}}{\sum_{i=1}^{N_{m}}m_{i}^{\mathrm{eff}}\left(l\right)},$$

where $N_{m}$ is the number of modes included in the analysis. In this study, the first six modes are considered. A larger positive value of R(l) indicates a greater effective-modal-mass-weighted reduction in generalized stiffness for the specified excitation direction.

## References

[1] Sun Z., Jayasinghe S., Sidiq A., Shahrivar F., Mahmoodian M., Setunge S. (2025). Approach Towards the Development of Digital Twin for Structural Health Monitoring of Civil Infrastructure: A Comprehensive Review. Sensors.

[2] Sarfarazi S., Shamass R., Rabi M., Abarkan I., Ferreira F.P.V., Tsavdaridis K.D. (2026). Inverse machine learning for the design of perforated beams: Parent section and material prediction. Eng. Appl. Artif. Intell..

[3] Dessena G., Civera M., Zanotti Fragonara L., Ignatyev D.I., Whidborne J.F. (2023). A Loewner-Based System Identification and Structural Health Monitoring Approach for Mechanical Systems. Struct. Control Health Monit..

[4] Deng K., Ompusunggu A.P., Xu Y., Skote M., Zhao Y. (2025). A Review of Material-Related Mechanical Failures and Load Monitoring-Based Structural Health Monitoring (SHM) Technologies in Aircraft Landing Gear. Aerospace.

[5] Feng D.C., Ding J.Y., Xie S.C., Li Y., Akiyama M., Lu Y., Beer M., Li J. (2024). Climate Change Impacts on the Risk Assessment of Concrete Civil Infrastructures. ASCE OPEN Multidiscip. J. Civ. Eng..

[6] Liew J., Rashidi M., Le K., Nazar A.M., Sorooshnia E. (2025). Reviewing a Decade of Structural Health Monitoring in Footbridges: Advances, Challenges, and Future Directions. Remote Sens..

[7] Gillich G.R., Furdui H., Abdel Wahab M., Korka Z.I. (2019). A robust damage detection method based on multi-modal analysis in variable temperature conditions. Mech. Syst. Signal Process..

[8] Gillich G.R., Praisach Z.I. (2014). Modal identification and damage detection in beam-like structures using the power spectrum and time–frequency analysis. Signal Process..

[9] Gillich G.R., Maia N.M.M., Wahab M.A., Tufisi C., Korka Z.I., Gillich N., Pop M.V. (2021). Damage Detection on a Beam with Multiple Cracks: A Simplified Method Based on Relative Frequency Shifts. Sensors.

[10] Gunes B., Gunes O. (2021). Vibration-Based Damage Evaluation of a Reinforced Concrete Frame Subjected to Cyclic Pushover Testing. Shock Vib..

[11] Hu W.H., Moutinho C., Caetano E., Magalhães F., Cunha Á. (2012). Continuous dynamic monitoring of a lively footbridge for serviceability assessment and damage detection. Mech. Syst. Signal Process..

[12] Liang Y., Li D., Song G., Feng Q. (2018). Frequency Co-integration-based damage detection for bridges under the influence of environmental temperature variation. Measurement.

[13] Maes K., Van Meerbeeck L., Reynders E.P.B., Lombaert G. (2022). Validation of vibration-based structural health monitoring on retrofitted railway bridge KW51. Mech. Syst. Signal Process..

[14] Peeters B., Maeck J., Roeck G.D. (2001). Vibration-based damage detection in civil engineering: Excitation sources and temperature effects. Smart Mater. Struct..

[15] Sha G., Radzieński M., Cao M., Ostachowicz W. (2019). A novel method for single and multiple damage detection in beams using relative natural frequency changes. Mech. Syst. Signal Process..

[16] Wah W.S.L., Chen Y.T., Owen J.S. (2021). A regression-based damage detection method for structures subjected to changing environmental and operational conditions. Eng. Struct..

[17] Chen H., Kurt M., Lee Y.S., McFarland D.M., Bergman L.A., Vakakis A.F. (2014). Experimental system identification of the dynamics of a vibro-impact beam with a view towards structural health monitoring and damage detection. Mech. Syst. Signal Process..

[18] Mirtaheri M., Salkhordeh M., Mohammadgholiha M. (2021). A System Identification-Based Damage-Detection Method for Gravity Dams. Shock Vib..

[19] Randiligama S., Thambiratnam D.P., Chan T.H.T., Fawzia S., Nguyen K.D. (2021). Vibration based damage detection in hyperbolic cooling towers using coupled method. Eng. Fail. Anal..

[20] Wang N.G., Jiang Y.Y., Zhong Y.T., Shao L. (2021). An adaptive damage detection method based on differential evolutionary algorithm for beam structures. Measurement.

[21] Altunışık A.C., Okur F.Y., Karaca S., Kahya V. (2019). Vibration-based damage detection in beam structures with multiple cracks: Modal curvature vs. modal flexibility methods. Nondestruct. Test. Eval..

[22] Nick H., Aziminejad A. (2021). Vibration-Based Damage Identification in Steel Girder Bridges Using Artificial Neural Network Under Noisy Conditions. J. Nondestruct. Eval..

[23] Yang Z.B., Radzienski M., Kudela P., Ostachowicz W. (2017). Fourier spectral-based modal curvature analysis and its application to damage detection in beams. Mech. Syst. Signal Process..

[24] Nick H., Aziminejad A., Hosseini M.H., Laknejadi K. (2021). Damage identification in steel girder bridges using modal strain energy-based damage index method and artificial neural network. Eng. Fail. Anal..

[25] Mao H., Tang W., Huang Y., Yuan D., Huang Z., Li X., Zheng W., Ma S. (2018). The construction and comparison of damage detection index based on the nonlinear output frequency response function and experimental analysis. J. Sound Vib..

[26] Medeiros R., Souza G.S.C., Marques D.E.T., Flor F.R., Tita V. (2021). Vibration-based structural monitoring of bi-clamped metal-composite bonded joint: Experimental and numerical analyses. J. Adhes..

[27] Padil K.H., Bakhary N., Abdulkareem M., Li J., Hao H. (2020). Non-probabilistic method to consider uncertainties in frequency response function for vibration-based damage detection using Artificial Neural Network. J. Sound Vib..

[28] Feng L., Yi X., Zhu D., Xie X., Wang Y. (2015). Damage detection of metro tunnel structure through transmissibility function and cross correlation analysis using local excitation and measurement. Mech. Syst. Signal Process..

[29] Hanif M.U., Ibrahim Z., Jameel M., Ghaedi K., Hashim H. (2021). Simulation-based non-linear vibration model for damage detection in RC beams. Eur. J. Environ. Civ. Eng..

[30] Peng Z., Li J., Hao H., Li C. (2021). Nonlinear structural damage detection using output-only Volterra series model. Struct. Control Health Monit..

[31] Huang T.X., Schroder K.U. (2021). A Bayesian probabilistic approach for damage identification in plate structures using responses at vibration nodes. Mech. Syst. Signal Process..

[32] Huang T.X., Schroder K.U. (2021). IWSHM 2019: Perturbation-based Bayesian damage identification using responses at vibration nodes. Struct. Health Monit..

[33] Jahangiri M., Hadianfard M.A., Najafgholipour M.A., Jahangiri M. (2021). A reliability-based sieve technique: A novel multistage probabilistic methodology for the damage assessment of structures. Eng. Struct..

[34] Ji X., Fenves G.L., Kajiwara K., Nakashima M. (2011). Seismic Damage Detection of a Full-Scale Shaking Table Test Structure. J. Struct. Eng..

[35] Kang F., Li J.j., Xu Q. (2012). Damage detection based on improved particle swarm optimization using vibration data. Appl. Soft Comput..

[36] Luca F., Manzoni S., Cigada A., Frate L. (2022). A vibration-based approach for health monitoring of tie-rods under uncertain environmental conditions. Mech. Syst. Signal Process..

[37] Mariniello G., Pastore T., Menna C., Festa P., Asprone D. (2021). Structural damage detection and localization using decision tree ensemble and vibration data. Comput.-Aided Civ. Infrastruct. Eng..

[38] Naderi A., Sohrabi M.R., Ghasemi M.R., Dizangian B. (2021). A swift technique for damage detection of determinate truss structures. Eng. Comput..

[39] Pepi C., Cavalagli N., Gusella V., Gioffre M. (2021). Damage detection via modal analysis of masonry structures using shaking table tests. Earthq. Eng. Struct. Dyn..

[40] Wickramasinghe W.R., Thambiratnam D.P., Chan T.H.T., Nguyen T. (2016). Vibration characteristics and damage detection in a suspension bridge. J. Sound Vib..

[41] Chang K.C., Kim C.W. (2016). Modal-parameter identification and vibration-based damage detection of a damaged steel truss bridge. Eng. Struct..

[42] Pereira S., Magalhaes F., Gomes J.P., Cunha A., Lemos J.V. (2021). Vibration-based damage detection of a concrete arch dam. Eng. Struct..

[43] Zhang F.L., Kim C.W., Goi Y. (2021). Efficient Bayesian FFT method for damage detection using ambient vibration data with consideration of uncertainty. Struct. Control Health Monit..

[44] Dessena G., Civera M., Pontillo A., Ignatyev D.I., Whidborne J.F., Zanotti Fragonara L. (2024). Noise-robust modal parameter identification and damage assessment for aero-structures. Aircr. Eng. Aerosp. Technol..

[45] Dessena G., Civera M. (2025). Improved tangential interpolation-based multi-input multi-output modal analysis of a full aircraft. Eur. J. Mech. A/Solids.

[46] Izadi A., Esfandiari A. (2024). Finite element model updating for structural damage detection using transmissibility data. Earthq. Eng. Eng. Vib..

[47] Bao Y., Li H. (2021). Machine learning paradigm for structural health monitoring. Struct. Health Monit..

[48] Zeng J., Kim Y.H. (2022). Probabilistic damage detection and identification of coupled structural parameters using Bayesian model updating with added mass. J. Sound Vib..

[49] Hou R., Xia Y. (2021). Review on the new development of vibration-based damage identification for civil engineering structures: 2010–2019. J. Sound Vib..

[50] Serlenga V., Gallipoli M.R., Ditommaso R., Ponzo C.F., Tragni N., Perrone A., Stabile T.A., Calamita G., Vignola L., Carso R.F. (2021). An integrated approach for structural behavior characterization of the Gravina Bridge (Matera, Southern Italy). Struct. Health Monit..

[51] Kim C.W., Zhang F.L., Chang K.C., McGetrick P.J., Goi Y. (2021). Ambient and Vehicle-Induced Vibration Data of a Steel Truss Bridge Subject to Artificial Damage. J. Bridge Eng..

[52] Zhang Y.K., Wei S.Y., Huang Y., Su Y.W., Lu S.S., Jiang K., Li H. (2025). SDIGLM: Leveraging large language models and multimodal chain of thought for structural damage identification. Struct. Health Monit. Int. J..

[53] Yu Y., Liu Y. (2024). Physics-guided generative adversarial network for probabilistic structural system identification. Expert Syst. Appl..

[54] Li Y.T., Bao T.F., Gao Z.X., Shu X.S., Zhang K., Xie L.C., Zhang Z.T. (2022). A new dam structural response estimation paradigm powered by deep learning and transfer learning techniques. Struct. Health Monit. Int. J..

[55] Huang L., Zuo Y., Guo C., Wang B., Tian K. (2025). Machine learning in flight parameter-based structural load prediction: A review and framework proposal. Prog. Aerosp. Sci..

[56] Wang Z.L., Cha Y.J. (2025). Unsupervised machine and deep learning methods for structural damage detection: A comparative study. Eng. Rep..

[57] Wang Z., Khokhar S.A., Jahanshahi M.R., Fu Y., Bilionis I., Maghareh A., Dyke S.J. (2026). Unsupervised anomaly detection based on deep autoencoders, information fusion, and active sensing. Struct. Health Monit..

[58] Wang Z., Jahanshahi M.R., Azimi M., Dyke S.J. (2024). Sensor Fault Detection in Smart Extraterrestrial Habitats Using Unsupervised Learning. AIAA J..

[59] Gunes B. (2022). One-class machine learning approach for localized damage detection. J. Civ. Struct. Health Monit..

[60] Bisheh H.B., Amiri G.G. (2023). Structural damage detection based on variational mode decomposition and kernel PCA-based support vector machine. Eng. Struct..

[61] Rafiei M.H., Adeli H. (2018). A novel unsupervised deep learning model for global and local health condition assessment of structures. Eng. Struct..

[62] Soleimani-Babakamali M.H., Soleimani-Babakamali R., Sarlo R., Farghally M.F., Lourentzou I. (2023). On the effectiveness of dimensionality reduction for unsupervised structural health monitoring anomaly detection. Mech. Syst. Signal Process..

[63] Seventekidis P., Giagopoulos D. (2023). Model error effects in supervised damage identification of structures with numerically trained classifiers. Mech. Syst. Signal Process..

[64] Zahoor R., Catalano E., Vallifuoco R., Zeni L., Minardo A. (2023). Automated Damage Detection Using Lamb Wave-Based Phase-Sensitive OTDR and Support Vector Machines. Sensors.

[65] Oliveira J.G.P., Sotelino E.D. (2025). Damage detection in reinforced concrete bridge: An ANN-based approach for inspection. Struct. Infrastruct. Eng..

[66] Pan S.J., Yang Q. (2010). A Survey on Transfer Learning. IEEE Trans. Knowl. Data Eng..

[67] Heravi M.A., Soleimani-Babakamali M.H., Naderpour H., Sadhu A. (2026). Zero-shot transfer learning for structural damage detection using target-to-source structure domain data mapping. Mech. Syst. Signal Process..

[68] Mariniello G., Pastore T., Asprone D. (2025). Spectral Jump Anomaly Detection: Temperature-compensated algorithm for structural damage detection using vibration data. Autom. Constr..

[69] Wan K., Wang Y., Wang J. (2025). Structural damage localization: A prior knowledge-based unsupervised approach using multimodal convolutional neural networks. Measurement.

[70] Zhao Z., Chen N.Z. (2024). A single-sensor method for structural damage localization in wind turbine blades: Laboratory assessment on a blade segment. Mech. Syst. Signal Process..

[71] Garrido H., Domizio M., Curadelli O., Ambrosini D. (2020). Numerical, statistical and experimental investigation on damage quantification in beams from modal curvature. J. Sound Vib..

[72] Das S., Roy K. (2024). Frequency response function-based closed-form expression for multi-damage quantification and its application on shear buildings. Mech. Syst. Signal Process..

[73] Karniadakis G.E., Kevrekidis I.G., Lu L., Perdikaris P., Wang S., Yang L. (2021). Physics-informed machine learning. Nat. Rev. Phys..

[74] Willard J., Jia X., Xu S., Steinbach M., Kumar V. (2022). Integrating Scientific Knowledge with Machine Learning for Engineering and Environmental Systems. ACM Comput. Surv..

[75] Hu Z., Xiang Z., Lu Q. (2020). Passive Tap-scan damage detection method for beam structures. Struct. Control Health Monit..

[76] Ikeda T., Yasui S., Minamiyama S., Ohara K., Ashizawa S., Ichikawa A., Okino A., Oomichi T., Fukuda T. (2018). Stable impact and contact force control by UAV for inspection of floor slab of bridge. Adv. Robot..

[77] Alazmi Y.H., Al-Zu’bi M., Al-Kheetan M.J., Rabi M. (2025). A Review of Robotic Applications in the Management of Structural Health Monitoring in the Saudi Arabian Construction Sector. Buildings.

[78] Nagarajaiah S., Basu B. (2009). Output only modal identification and structural damage detection using time frequency & wavelet techniques. Earthq. Eng. Eng. Vib..

[79] Ching J., Chen Y.C. (2007). Transitional Markov Chain Monte Carlo Method for Bayesian Model Updating, Model Class Selection, and Model Averaging. J. Eng. Mech..

[80] Wang Z., Jahanshahi M.R., Lund A., Shahriar A., Montoya A. (2025). Physics-Informed Machine Learning for Hybrid Digital Twin–Enhanced Damage Detection and Localization. J. Eng. Mech..

[81] Chen L., Chen H., Wei Z., Jin X., Tan X., Jin Y., Chen E. Reusing the Task-Specific Classifier as a Discriminator: Discriminator-Free Adversarial Domain Adaptation. Proceedings of the IEEE/CVF Conference on Computer Vision and Pattern Recognition (CVPR).

[82] Avitabile P. (2017). Introduction to Experimental Modal Analysis: A Simple Non-mathematical Presentation. Modal Testing.

[83] Jia X., Willard J., Karpatne A., Read J.S., Zwart J.A., Steinbach M., Kumar V. (2021). Physics-Guided Machine Learning for Scientific Discovery: An Application in Simulating Lake Temperature Profiles. ACM/IMS Trans. Data Sci..

[84] Ganin Y., Ustinova E., Ajakan H., Germain P., Larochelle H., Laviolette F., March M., Lempitsky V. (2016). Domain-adversarial training of neural networks. J. Mach. Learn. Res..

[85] Maia N.M., Almeida R.A., Urgueira A.P., Sampaio R.P. (2011). Damage detection and quantification using transmissibility. Mech. Syst. Signal Process..

[86] Johnson E.A., Lam H.F., Katafygiotis L.S., Beck J.L. (2004). Phase I IASC-ASCE Structural Health Monitoring Benchmark Problem Using Simulated Data. J. Eng. Mech..

[87] Dyke S.J., Bernal D., Beck J., Ventura C. (2003). Experimental phase II of the structural health monitoring benchmark problem. Proceedings of the 16th ASCE Engineering Mechanics Conference.

[88] Amin A.A.H., Aladdin A.M., Hasan D.O., Mohammed-Taha S.R., Rashid T.A. (2023). Enhancing Algorithm Selection through Comprehensive Performance Evaluation: Statistical Analysis of Stochastic Algorithms. Computation.

[89] Zhou X., Kim C.W., Zhang F.L., Chang K.C. (2022). Vibration-based Bayesian model updating of an actual steel truss bridge subjected to incremental damage. Eng. Struct..

